# Transcriptome and Biochemical Analysis of a Flower Color Polymorphism in *Silene littorea* (Caryophyllaceae)

**DOI:** 10.3389/fpls.2016.00204

**Published:** 2016-02-29

**Authors:** Inés Casimiro-Soriguer, Eduardo Narbona, M. L. Buide, José C. del Valle, Justen B. Whittall

**Affiliations:** ^1^Department of Molecular Biology and Biochemical Engineering, Pablo de Olavide UniversitySeville, Spain; ^2^Department of Plant Biology and Ecology, University of SevilleSeville, Spain; ^3^Department of Biology, Santa Clara University, College of Arts and SciencesSanta Clara, CA, USA

**Keywords:** *Silene littorea*, anthocyanin biosynthetic pathway, flavonoid biochemistry, mRNA-Seq, HPLC, flower color polymorphism, transcriptome, flavanone-3-hydroxylase

## Abstract

Flower color polymorphisms are widely used as model traits from genetics to ecology, yet determining the biochemical and molecular basis can be challenging. Anthocyanin-based flower color variations can be caused by at least 12 structural and three regulatory genes in the anthocyanin biosynthetic pathway (ABP). We use mRNA-Seq to simultaneously sequence and estimate expression of these candidate genes in nine samples of *Silene littorea* representing three color morphs (dark pink, light pink and white) across three developmental stages in hopes of identifying the cause of flower color variation. We identified 29 putative paralogs for the 15 candidate genes in the ABP. We assembled complete coding sequences for 16 structural loci and nine of ten regulatory loci. Among these 29 putative paralogs, we identified 622 SNPs, yet only nine synonymous SNPs in *Ans* had allele frequencies that differentiated pigmented petals (dark pink and light pink) from white petals. These *Ans* allele frequency differences were further investigated with an expanded sequencing survey of 38 individuals, yet no SNPs consistently differentiated the color morphs. We also found one locus, *F3h1*, with strong differential expression between pigmented and white samples (>42x). This may be caused by decreased expression of *Myb1a* in white petal buds. *Myb1a* in *S. littorea* is a regulatory locus closely related to Subgroup 7 Mybs known to regulate *F3h* and other loci in the first half of the ABP in model species. We then compare the mRNA-Seq results with petal biochemistry which revealed cyanidin as the primary anthocyanin and five flavonoid intermediates. Concentrations of three of the flavonoid intermediates were significantly lower in white petals than in pigmented petals (rutin, quercetin and isovitexin). The biochemistry results for rutin, quercetin, luteolin and apigenin are consistent with the transcriptome results suggesting a blockage at *F3h*, possibly caused by downregulation of *Myb1a*.

## Introduction

Flower color has played a pivotal role in our current understanding of biology since Mendel's discovery of the inheritance of flower color in *Pisum sativum* (Mendel, 1866; Ellis et al., 2011). Since then, flower color has contributed to a wide range of important biological discoveries including gene regulation (Napoli et al., 1990), pleiotropy (Streisfeld and Rausher, 2011), population genetics (Wright, 1943; Schemske and Bierzychudek, 2001), speciation (Bradshaw et al., 1995; Hopkins and Rausher, 2011) and floral ecology (Irwin and Strauss, 2005; Eckhart et al., 2006; Strauss and Whittall, 2006). Although many breakthroughs involving flower color utilize model species with available complete reference genomes, numerous evolutionary and ecological questions regarding flower color variation reside in non-model species. Investigating the cause of flower color variation in non-model species would benefit from an efficient method for sequencing and detecting expression of all flower color related genes in non-model species.

The most common floral pigments are the anthocyanins (Miller et al., 2011; Campanella et al., 2014) which are produced by the anthocyanin biosynthetic pathway (ABP). Floral anthocyanins are now considered a metamodel because of the conserved nature of the biosynthetic pathway across most angiosperms (Kopp, 2009). Changes in the color of anthocyanins (e.g., shifts from blue to red flowers) and the loss of floral anthocyanins (producing white flowers) can now be traced from ecological interactions in the field to the biochemical and molecular basis for these changes (Tanaka et al., 2008; Davies, 2009; Hopkins and Rausher, 2011; Zhao and Tao, 2015). These changes can result from mutations in core structural genes or regulatory loci (Sobel and Streisfeld, 2013). It is expected that null coding mutations will be more frequent within species; and cis-regulatory mutations between species (Stern and Orgogozo, 2008), which has been demonstrated in polymorphic populations of some species such as *Mimulus lewisii* (Wu et al., 2013).

The ABP is composed of seven core enzymes and several side-branching enzymes, and appears largely conserved across angiosperms (Holton and Cornish, 1995; Grotewold, 2006). Blockages in the first steps of the ABP are predicted to have more dramatic physiological effects and potentially ecological consequences compared to blockages in the latter steps because of the lack of stress-responsive flavones and flavonols (Whittall et al., 2006). These maladaptive consequences of blocking the ABP can be ameliorated by recruiting tissue-specific regulators in order to limit effects solely to the petal (Streisfeld and Rausher, 2011; Wessinger and Rausher, 2012; Sobel and Streisfeld, 2013). The ABP is regulated by a complex composed by three interacting transcription factors: the R2R3-MYB, the basic helix-loop-helix (bHLH) and the WD40-repeat (WDR) (Hichri et al., 2011). The MYBs confer the majority of the tissue specificity (Zhang et al., 2003; Schwinn et al., 2006; Dubos et al., 2010; reviewed in Albert et al., 2014). Collectively, the core ABP, side-branches within the ABP, genes leading into the ABP and regulatory genes provides a relatively large target for a diversity of mutations that could block the ABP (Wessinger and Rausher, 2012). For flower color polymorphic plants, locating the blockage and predicting the physiological and ecological consequences require a thorough characterization of the ABP at the biochemical and molecular scales (e.g., Lou et al., 2014; Nishihara et al., 2014). Deciphering the cause of flower color variation is a complicated task because it requires sequencing and measuring expression of all genes acting in the ABP and their regulators.

RNA-Seq is a fast and efficient approach to sequence and examine the expression of all ABP-related genes, even when a reference genome is not available as in most non-model species (Li et al., 2012; Lulin et al., 2012; Xu et al., 2013; Butler et al., 2014). For flower color polymorphisms, petal mRNA must be examined across a range of developmental stages, especially the earliest stage when the flower color polymorphism is first manifested (Whittall et al., 2006; Dick et al., 2011; Butler et al., 2014). Large, complex genomes often exhibit multiple paralogs, sometimes expressed in the same tissue (e.g., Martins et al., 2013; Yuan et al., 2014). Differentiating paralogs and getting paralog-specific expression levels can be a complicated step in the mRNA-Seq bioinformatics pipeline. Once a candidate gene has been identified with either sequence or expression differences that correlate with flower color, subsequent biochemical analysis of the petal can be used to test the flavonoid composition. A blockage in the ABP should restrict the production of downstream flavonoids and may lead to an accumulation of upstream intermediates (Whittall et al., 2006; Dick et al., 2011). High-Performance Liquid Chromatography (HPLC) coupled with mass spectrometry has been extensively used to identify and quantify the flavonoid composition in many ornamental and wild plants (Fossen and Andersen, 2006; Qiao et al., 2011). For instance, high concentrations of anthocyanins in black cultivars of *Dahlia*, were related with elevated expression of the ABP genes and low concentrations of flavones (Thill et al., 2012).

**Figure 1:**
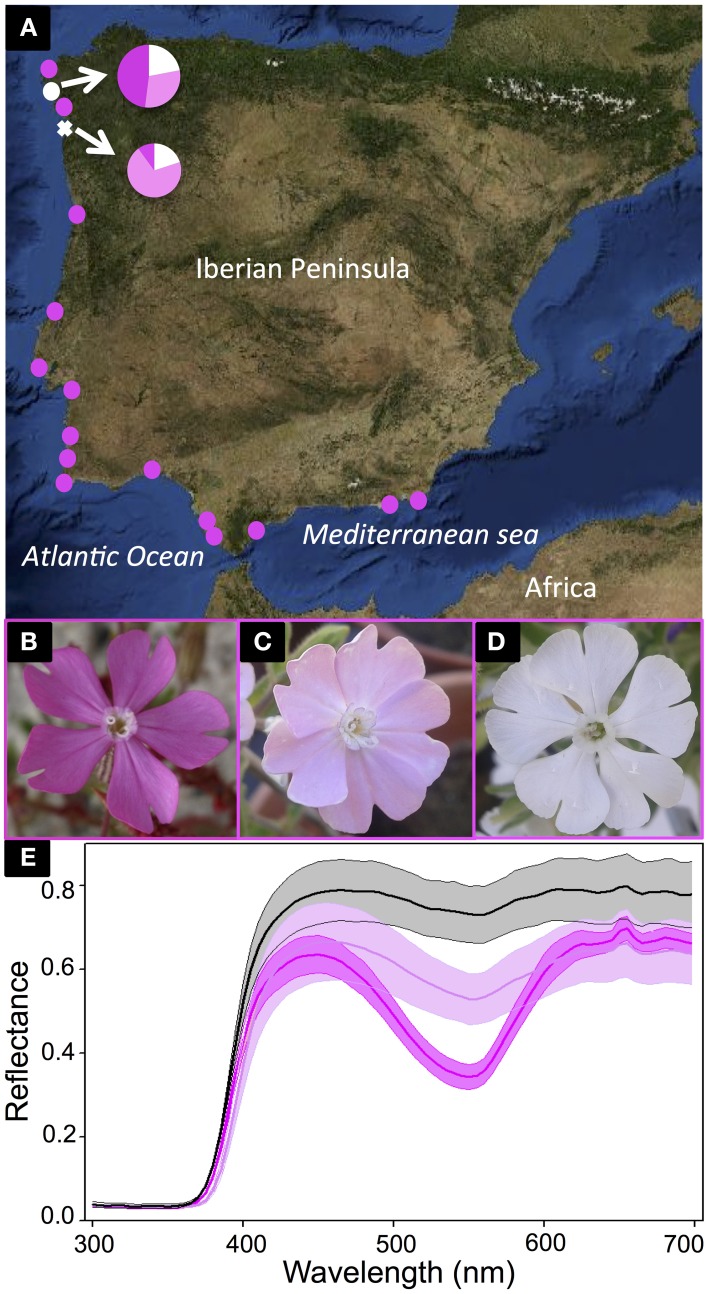
*****Silene littorea*** sampling and flower color polymorphism**. The map in **(A)** shows *S. littorea* populations along the Iberian Peninsula where flower color frequencies were estimated. The two polymorphic populations are indicated with white symbols and the proportions of white-, light pink-, and dark pink-flowered individuals in each population are illustrated with pie diagrams. Pink circles indicate populations fixed for dark pink petal color. The polymorphic population (Playa de Barra) sampled for RNA-Seq and biochemical analysis is indicated with a white cross. The three distinct color morphs are illustrated in **(B–D)**: dark pink **(B)**, light pink **(C)**, and white **(D)**. The average UV-VIS spectral reflectance of the upper surface of the petals of six dark pink samples (dark pink line), six light pink samples (light pink line), and three white samples (black line) are indicated in **(E)** with standard errors (shadow area).

The genus *Silene* (Caryophyllaceae) is a model for studies of evolutionary ecology (Bernasconi et al., 2009), yet no one has examined the molecular and biochemical basis for flower color polymorphisms in any of the species (yet see the proposed ABP in Kamsteeg et al., 1979). Although the Caryophyllales are largely characterized by the production of betalain pigments in place of anthocyanins, flower color variation in *Silene* is still caused by anthocyanins (Brockington et al., 2011). Herein, we focus on a discrete flower color polymorphism in the Iberian Peninsula endemic, *S. littorea* (Talavera, 1979). After surveying 17 populations across the species range, we found most populations were composed of primarily dark pink individuals (Figure 1A). Yet, in two northern populations, there were three distinct color morphs: white, light pink, and dark pink (Figures 1B–D). The three petal color morphs were compared with a UV-Vis spectrophotometer. The differences among them are concentrated in the visible range especially at the typical wavelength that anthocyanins absorb (~550 nm; Figure 1E), yet the biochemical and molecular underpinnings causing this flower color variation in *S. littorea* remains unknown.

Here, we examine the petal transcriptome and biochemistry of the flower color polymorphism in *S. littorea* using RNA-Seq complemented with HPLC flavonoid profiling to try and identify the most likely cause of the blockage in the ABP. Transcriptome analysis is used to sequence and estimate expression of 15 ABP-related genes. The sequences of these candidates are examined to determine if there are any consistently color differentiating SNPs. Simultaneously, we estimate expression differences between color morphs to establish if downregulation of any ABP-related genes correlate with white petals. We complement our RNA-Seq results with an investigation of the petal biochemistry of the three color morphs by identifying the primary anthocyanin pigment and flavonoid intermediates. We then compare their relative abundances among the three color morphs to help target the blockage in the ABP leading to white petals. The biochemical results are interpreted in light of the SNP and expression findings from the transcriptome analysis.

## Materials and methods

### Plant species

*Silene littorea* (Caryophyllaceae) has an anthocyanin petal polymorphism with three distinct categories—dark pink (D), light pink (L), and white (W) (Figures 1B–D). It belongs to the section *Psammophilae* (Oxelman et al., 2013), that is composed of five diploid (2*n* = 24; Talavera, 1979), annual, primarily pink flowered taxa. The species grows primarily in coastal sand dunes of Spain and Portugal (Figure 1A; Talavera, 1979). It has a gynodioecious-gynomonoecious sexual system and produces a highly variable number of flowers per plant (Casimiro-Soriguer et al., 2013), yet the flower color is constant among flowers within a plant (unpublished observations). There is no correlation between flower color and sexuality (unpublished observations), but white individuals are much more common in the northwestern portion of the species range compared to the southeast (Figure 1A).

### Sampling and RNA extraction

Plants were sampled from a polymorphic population near the northwestern range limit (Playa de Barra, Spain; 42° 15′ 35″N, 8° 50′ 25″W) (Figure 1A). Petals from W, L, and D flowers were sampled at three developmental stages: bud, opening, and anthesis (Figure S1 in Supplementary Material). All five petals from the same flower were collected, immediately preserved in RNA*later* (Ambion, Inc., Austin, Texas), and stored at −20°C until RNA extraction. RNA was isolated using the RNeasy Plant Mini Kit (Qiagen, Valencia, CA). Concentration and purity of RNA was measured with a NanoDrop ND-1000 spectrophotometer (NanoDrop Technologies, Inc., Wilmington, DE) and agarose gels were run to verify RNA integrity. The nine samples with the highest concentration of RNA for each of the three color morphs and developmental stages were selected: bud white, opening white, anthesis white, bud light, opening light, anthesis light, bud dark, opening dark, and anthesis dark.

### Library preparation and sequencing

RNA-Seq libraries were prepared and sequenced at the Epigenome Center of the University of Southern California following the manufacturers protocol (Illumina, San Diego, CA). The nine libraries were barcoded (6 bp), pooled in equimolar concentrations and loaded on a single lane of the Illumina Hi-Seq 2000 system. Sequencing consisted of 50 cycles of single-end sequencing-by-synthesis reactions. Fortuitously, the libraries were sequenced twice. After a preliminary analysis indicated that both datasets produced qualitatively similar results, we merged them for all analyses reported herein. In the combined dataset, there was an average of 37.9 million reads per sample (range 36.3–40.0 million). Raw sequencing reads were deposited to NCBI's Short Read Archive (Accession number SRP033277).

### *De novo* assembly of ABP-related loci

Since there are no closely related genome resources for *Silene*, we conducted *de novo* assembly of the *S. littorea* transcriptome following a similar pipeline developed by Butler et al. (2014) relying largely on VELVET v.1.2.07 (Zerbino and Birney, 2008) and OASES v.0.2.08 (Schulz et al., 2012). We assembled using the FASTQ files across a range of *k*-mers (23-39) to maximize ABP candidate gene coverage. Assembled contigs were identified by BLAST+ against the *Arabidopsis thaliana* RefSeq database from TAIR (v. 10; Swarbreck et al., 2008), limiting the results to *e* < 10^−10^.

We made sequence comparisons and expression analyses of 15 ABP candidate genes. These include seven core ABP structural genes [chalcone synthase (*Chs*), chalcone isomerase (*Chi*), flavanone-3-hydroxylase (*F3h*), dihydroflavonol 4-reductase (*Dfr*), anthocyanidin synthase (*Ans*), flavonoid 3-O-glucosyltransferase (*Uf3gt*), and acyltransferase (*At*)], three genes immediately upstream of the ABP [phenylalanine ammonia-lyase (*Pal*), cinnamate 4-hydroxylase (*C4h*), coumarate CoA ligase (*4Cl*)], two side-branching genes [flavonol synthase (*Fls*), flavonoid 3′hydroxylase (*F3*′*h*)], and three transcriptional regulators [basic helix loop helix (*Bhlh*), WD40 Repeats (*Wd40*), and R2R3-MYB domains (*Mybs*)]. For each of the nine samples, we extracted all contigs from the candidates for each gene of the ABP and these contigs were aligned in BioEdit (Hall, 1999). For two regulatory genes, *Wd40* and *Mybs*, two and seven different sequences were found. For five core ABP genes, two to three very distinct sequences of the same gene were assembled. Even though these loci blasted to the same ABP-related locus in the TAIR database, the alignment suggested they were unlikely orthologous. Therefore, we treated them separately as putative paralogs (hereafter “locus”) in all further analyses of 29 loci in total. For each locus, a consensus sequence with ambiguities representing all the variable sites among the nine samples was extracted as the reference for gene expression. All ABP-related reference loci are available in Genbank nucleotide database [http://www.ncbi.nlm.nih.gov/nuccore/] Accession numbers KT954895-KT954923.

### Sequence comparisons of ABP-related loci

For all 29 ABP related loci, we tested for single nucleotide polymorphisms (SNPs) that correlated with flower color. We started by mapping the microreads back onto the *de novo* consensus sequences using Mosaik (Lee et al., 2014). We followed the author's recommendations for the parameter settings: two allowed mismatches and a hash length of 15. We then used Picard v.1.94 (http://broadinstitute.github.io/picard/) to identify PCR duplicate reads—an artifact of the library preparation methodology. The Genome Analysis Toolkit v.2.6-4 (GATK, McKenna et al., 2010) was used to (1) re-align the reads around potential indels, (2) remove the PCR duplicates identified in Picard and finally (3) identify SNPs in each of the nine samples across the 29 ABP-related loci (DePristo et al., 2011). We calculated the allele frequencies for each SNP for each color type using the genotype field (GT) in the GATK output file (we surveyed 3 individuals per color morph = 6 alleles per color morph). We also calculated the mean likelihood of genotype assignment (0/0, 0/1, or 1/1) for each color type (parameter PL in the GATK output).

After finding allele frequency differences among pink and white individuals in *Ans*, we conducted a survey of additional individuals from the same population using genomic DNA. DNA was extracted from 19 pink and 19 white individuals following the PL2 protocol in the NucleoSpin Plant II kit (Macherey-Nagel, Bethlehem, PA). We designed primers specific to *SlAns* in order to amplify and sequence a 677 bp fragment including the color differentiating SNPs and a 110 bp intron (ANS-416F: CTAGTGGCCAACTCGAGTGG & ANS-1021R: CAAAGGTTCGAGGCGGGTAA). PCR conditions followed those of Dick et al. (2011) using *Taq* polymerase from New England Biolabs (Ipswich, Massachusetts) with the following thermal cycling steps: initial denature at 95°C for 3 min; 35 cycles of 95°C for 30 s, 60°C for 30 s, 72°C for 90 s; a final extension at 72°C for 10 min; and a 4°C hold. PCR products were purified using exoSAP and sequenced using Big Dye Terminator methodology on an ABI 3730xl DNA Analyzer (Sequetech Corp., Mountain View, California). Contigs were produced from forward and reverse reads. Contigs were then aligned and allele frequencies calculated in Geneious v.8.1.6 (Auckland, New Zealand). The flower color of each additional sample in the *Ans* survey was verified with an anthocyanin extraction of the petals and quantification by measuring their absorbance at 520 nm (see Materials and Methods in Del Valle et al., 2015) following correction for the petal area (Figure S2 in Supplementary Material).

### Expression analysis

To extract the number of reads mapped for each gene from the bam file produced by Mosaik we used Artemis (Rutherford et al., 2000). For differential expression analysis we used the DESeq package (Anders and Huber, 2010) in R (R Core Team, 2013). This package requires the normalization of the raw counts. After normalization, only those loci above the 33rd quantile are further analyzed for differential expression. This filtering step is neccesary to avoid spurious estimates of fold-change differences due to very low expression values. A negative binomial tests was applied to identify any statistically differentially expressed loci (*p* < 0.05). Although we analyzed the three developmental stages separately, we focus the remainder of the expression analysis and interpretation on the bud stage since the petal color is already present in the bud (Figure S1 in Supplementary Material) and therefore, the causal genes should be detectable at this developmental stage.

### Phylogenetic analysis of R2R3 mybs

To help infer which *S. littorea* petal *Myb*s may be involved in anthocyanin biosynthesis, we compared the seven distinct *Mybs* identified in *S. littorea* to known regulators of the ABP from several model species (*Antirrhinum majus, Arabidopsis thaliana, Chrysanthemum morifolium, Eucaliptus gunnii, Fragaria ananassa, Fragaria chiloensis, Gerbera hybrida, Ipomoea nil, Lycopersicon esculentum, Malus domestica, Mimulus aurantiacus, Salvia miltiorrhiza, Oryza sativa, Petunia hybrida, Populus trichocarpa, Vitis vinifera*, and *Zea mays*—accession numbers can be found in Table S1 in Supplementary Material). We conducted both maximum likelihood (RAxML; Stamatakis, 2014) and Bayesian (MrBayes; Huelsenbeck and Ronquist, 2001) phylogenetic analyses of the aligned nucleotids of the R2R3 binding domain (315 bp) using plug-ins within Geneious v.8.1.6. For the maximum likelihood analysis, we fit a GTR+CAT+I model followed by 1000 bootstrap replications. For the Bayesian analysis, we applied the GTR+I+G model of sequence evolution for two separate runs, each consisting of four independent chains run for 5,000,000 generations sampling every 50,000 generations after 1,000,000 generations of burnin. Bayesian runs were checked for proper mixing and convergence using standard diagnostics.

### HPLC analysis of flavonoids

Petal flavonoids were identified for three dark pink samples. After that, we compared specific compounds in three white, six light pink, and six dark pink samples. Flavonoids were extracted from four petals of an anthesis stage flower that were preserved in 1 mL of CH_3_OH:H_2_O:HCl (90:9:1, v:v:v) and stored on ice and in the dark until the flavonoids could be extracted. The samples were homogenized using 5 × 3 mm diameter glass beads in a Mixer Mill MM 200 (Retsch, Haan, Germany) with a frequency of 30 oscillations/s. They were beaten until the sample was completely homogenized (minimum of 60 s). The supernatant was removed after 10 min centrifugation (13,000 rpm) and stored at −20°C until it could be separated by HPLC.

Chromatographic separation was performed using a Perkin Elmer Series 200 HPLC system (Wellesley, USA) coupled to an Applied Biosystems QTRAP LC/MS/MS system (Foster City, USA) consisting of a hybrid triple-quadruple linear ion trap (QqQLIT) mass spectrometer equipped with an electrospray ion source. HPLC analyses were performed on a 150 × 2.0 mm Phenomenex Luna 3u C18(2) 100A reversed-phase column with a particle size of three μm. The flow rate was 0.2 mL/min. To identify and quantify the flavonoid compounds in the petals of *S. littorea*, we performed multiple reactions monitoring (MRM) combined with precursor ions scan and subsequent MS/MS analysis (Li et al., 2006; Qiao et al., 2011). We used the standards of the flavonoids that were previously reported for *S. littorea* and others species of *Silene* (Table S2 in Supplementary Material). The standards were obtained from SDS (Toulouse, France). The parameters for the MRM transitions and HPLC-ESI-MS/MS analyses were fixed following Dardanelli et al. (2008), with the exception of the dwell time for each transition which was set to 0.05 s. Flavonoid amounts were corrected for flower size using the total area of the petals measured with the software ImageJ (US National Institutes of Health, Bethesda, MD, USA, http://imagej.nih.gov/ij/). Size-corrected flavonoid amounts were standardized by their maximum value. Significant differences in individual flavonoid concentrations were examined for the three color categories (D, L, and W) in an ANOVA after log transformation in R v.3.1.0 (R Core Team, 2013). When significant, we conducted Tukey HSD *post-hoc* paired tests to determine which color morphs exhibited significantly different mean flavonoid concentrations.

## Results

### *De novo* assembly of ABP-related genes

We identified all 15 ABP-related genes from *de novo* assembly of the petal transcriptome. The longest contigs from the *de novo* assembly were most frequently from Velvet *k*-mer 31 (Table 1), but supplemented by contigs from other kmer analyses as necessary. After BLAST+ identification against all known genes of *A. thaliana*, multiple putative paralogs were identified for seven genes producing a total of 29 ABP-related loci (Table 2). Three of the genes that feed into the ABP had two or three copies each (*Pal, C4h*, and *4Cl*). Most of the ABP structural genes and their side-branches had only a single locus expressed in the petals except *Chs* and *F3h* which had two and three copies respectively. Of the three regulatory loci, there were seven *Myb*s, two *Wd40*s, and only one *Bhlh* locus. The bHLH locus is closely related to the AN1 locus of *Petunia* and TT8 locus from *Arabidopsis*, both regulators of the ABP (see Figure S3 in Supplementary material). We sequenced 100% of the coding sequence (CDS) for 28 of the 29 ABP-related loci (all except *Myb5*). In addition, we acquired an average of 121 bp of the 5′ UTR sequence (range 35–451 bp) and 170 bp of the 3′ UTR (range 21-306) (Table 2).

**Table 1:** **Summary of sequencing and assembling results from kmer = 31**.

**Sample**	**No. of reads**	**No. of assembled transcripts**	**Total length of transcripts (bp)**	**Average length of transcripts (bp)**
BW	37,153,142	25,708	14,519,322	564.8
BL	38,063,355	35,654	9,928,394	278.5
BD	38,535,318	32,339	11,387,548	352.1
OW	36,959,208	26,367	15,006,014	569.1
OL	39,381,159	21,635	14,194,798	656.1
OD	36,319,118	26,538	12,394,184	467.0
AW	38,199,741	32,422	15,417,417	475.5
AL	40,004,007	22,091	15,444,979	699.2
AD	36,623,636	30,973	14,016,310	452.3

*B, bud; O, opening; A, anthesis; W, white; L, light pink; D, dark pink*.

**Table 2:** **ABP sequencing results. Complete CDS and partial UTRs were sequenced for all ABP-related loci except for *Myb5*. The number, location and type of single nucleotide polymorphisms (SNPs) are indicated for each locus**.

**Locus**	**5′ UTR length (bp)**	**CDS length (bp)**	**3′ UTR length (bp)**	**No. SNPs in 5′ UTR**	**No. SNPs in CDS**	**No. SNPs in 3′ UTR**	**Total No. SNPs**	**No. Non-synonymous SNPs**
*Pal1*	119	2154	234	6	75	10	91	7
*Pal2*	118	2106	221	0	34	5	39	3
*Pal3*	55	2148	84	0	27	0	27	11
*C4h1*	78	1521	237	1	11	2	14	2
*C4h2*	451	1191	21	4	10	1	15	1
*4Cl1*	88	1692	147	1	27	2	30	4
*4Cl2*	99	1677	75	1	37	1	39	5
*Chs1*	60	1176	87	0	38	1	39	1
*Chs2*	136	1176	169	0	2	0	2	1
*Chi*	114	717	279	0	11	1	12	1
*F3h1*	129	1098	198	0	0	0	0	-
*F3h2*	179	1083	105	0	1	0	1	0
*F3h3*	40	1083	114	1	25	1	27	4
*Dfr*	108	1059	207	1	7	2	10	1
*Ans*	115	1098	216	2	24	6	32	7
*Uf3gt*	78	1374	273	1	28	5	34	8
*At*	88	1494	189	3	21	3	27	5
*F3′h*	41	1539	306	0	36	3	39	5
*Fls*	157	1176	270	1	3	0	4	2
*Myb1a*	128	708	160	1	10	2	13	1
*MYB1b*	61	729	152	0	4	2	6	2
*Myb2*	160	879	141	0	19	1	20	1
*Myb3*	222	711	147	1	14	2	17	4
*Myb4*	113	1032	72	0	5	1	6	3
*Myb5*	190	^*^577	–	0	5	–	5	0
*Myb6*	143	750	94	0	9	1	10	4
*Wd401*	106	1053	306	2	9	4	15	2
*Wd402*	35	1023	60	0	1	0	1	0
*Bhlh*	54	1914	198	1	42	4	47	24

* *Partial coding sequence*.

### Sequence comparisons among color morphs

Among the nine samples, we found 622 SNPs in the 5′ UTR, CDS and 3′ UTR of the 29 ABP-related loci (Table 2). The number of SNPs per gene was highly variable, ranging from zero to 91 in *F3h1* and *Pal1*, respectively (Table 2). Although we found numerous non-synonymous SNPs in several loci, none of them consistently differentiated the three color morphs.

*Ans* was the only gene that had SNPs with allele frequencies consistently associated with flower color (Table S3 in Supplementary Material). A total of 32 SNPs were found in the 5′ UTR, CDS and 3′ UTR in *Ans*, yet nine of these between positions 697–1099 exhibited substantially different frequencies in dark pink vs. white samples (Figure S4 in Supplementary Material). Furthermore, the likelihood of being homozygous for one allele or the other was also strongly correlated with petal color (Figure S5 in Supplementary Material). There was a very low likelihood of heterozygosity at all nine of these SNPs for all three color morphs. Nevertheless, all of these color-differentiating SNPs were synonymous.

Additional sequencing of the 677 bp region (including the 110 bp intron) of *Ans* in 19 pink and 19 white individuals contained all of the color differentiated SNPs except the last one at bp 1099. Seventeen SNPs including two additional SNPs discovered in the intron were examined, however no single SNP consistently differentiated pink and white individuals (mean allele frequency = 0.21; Table 3).

**Table 3:** **Broader SNP survey for ***Ans***. Genomic DNA sequencing of 19 dark pink and 19 white individuals from the same population as the transcriptome sequencing (Barra) reveals some allele frequency differences. No single SNP consistently differentiates the color morphs**.

**CDS Site No**.	**Reference allele (Ref.)**	**Alternate allele (Alt.)**	**Pink Ref. Homozygotes**.	**Pink Hets**.	**Pink Alt. Homozygotes**.	**White Ref. Homozygotes**.	**White Hets**.	**White Alt. Homozygotes**.	**Pink Ref. Allele Freq**.	**White Ref. Allele Freq**.	**Allele Freq Diff. (P-W)**
Intron1^*^	G	A	17	1	0	14	3	1	0.97	0.86	0.11
Intron2	A	G	18	1	0	14	4	1	0.97	0.84	0.13
697	A	C	6	9	4	1	2	16	0.55	0.11	0.45
709	A	G	16	3	0	13	4	2	0.92	0.79	0.13
746	C	T	7	9	3	1	4	14	0.61	0.16	0.45
763	C	A	7	10	2	3	5	11	0.63	0.29	0.34
790	A	T	17	2	0	17	2	0	0.95	0.95	0.00
799	G	A	7	10	2	3	7	9	0.63	0.34	0.29
845	C	T	15	4	0	15	3	1	0.89	0.87	0.03
853	T	C	16	3	0	19	0	0	0.92	1.00	0.08
898	C	G	6	11	2	3	5	11	0.61	0.29	0.32
913	A	G	5	9	5	1	4	14	0.50	0.16	0.34
919	C	T	15	3	1	14	3	2	0.87	0.82	0.05
937	C	A	6	10	3	1	4	14	0.58	0.16	0.42
970	C	T	1	2	16	0	3	16	0.11	0.08	0.03
982	C	T	18	1	0	15	3	1	0.97	0.87	0.11
994	G	T	5	10	4	1	5	13	0.53	0.18	0.34

* *Sequence data was only available for 18 pink individuals for this site*.

### Expression comparisons among color morphs

Since petal anthocyanins are detectable in the bud stage (Figure S1 in Supplementary Material), we infer that all ABP-related loci should have been expressed by this developmental stage. Thus, we focus on the three bud stage samples for expression comparisons (expression values for later developmental stages are available in Table S4 in Supplementary Material). The DESeq corrected expression estimates for the bud stage ranged from 1459.8 reads—494,875.6 reads (median = 8713.9 reads; Table S4 in Supplementary Material).

**Figure 2:**
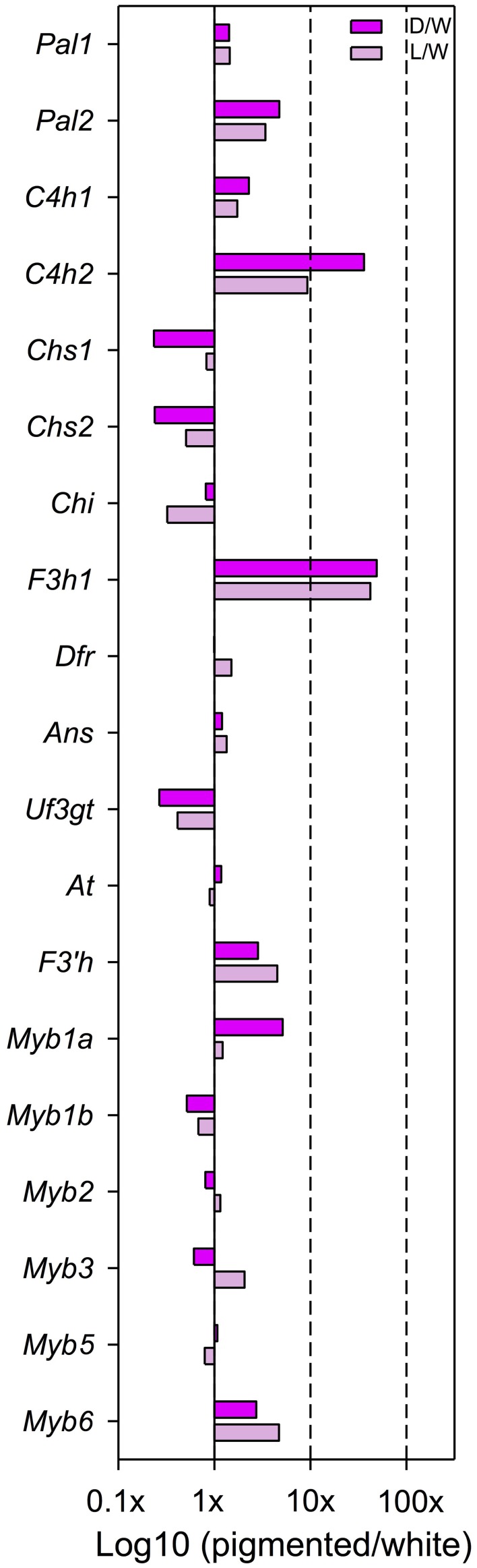
**Expression differences, estimated as fold changes, between dark/white (dark pink bars) and light/white (light pink bars) petals at bud developmental stage**. Fold change values between pigmented and white petals after normalization of the raw counts are shown. Only those loci that are above the lower 33 quantile are included. This filtering step prevents spourious estimates of fold-change values due to very low expression.

When comparing dark pink to white petal buds, there are three loci with significantly higher expression in dark pink than white: *F3h1* (D/W = 49.0x; p = 0.039), *C4h2* (D/W = 36.2x; *p* = 0.013), and *Myb1a* (D/W = 5.1x; *p* = 0.009) (Figure 2, Table S4 in Supplementary Material). When comparing light pink to white petal buds, there are two significantly differentially expressed loci: *F3h1* (L/W = 42.2x; p = 0.049) and *F3*′*h* (L/W = 4.5x; *p* = 0.047). Chalcone isomerase (*Chi*) is the only locus with W > L, yet only weakly so (L/W = 0.32x; *p* = 0.055 (Figure 2, Table S4 in Supplementary Material).

When comparing the two pigmented morphs (dark pink and light pink), there are two signficantly differentially expressed loci which are both regulatory—*Myb1a* (D/L = 4.2x; *p* = 0.021) and *Myb3* (D/L = 0.3; *p* = 0.033) (Table S4 in Supplementary Material).

### Phylogenetic analysis of R2R3 Myb loci

**Figure 3:**
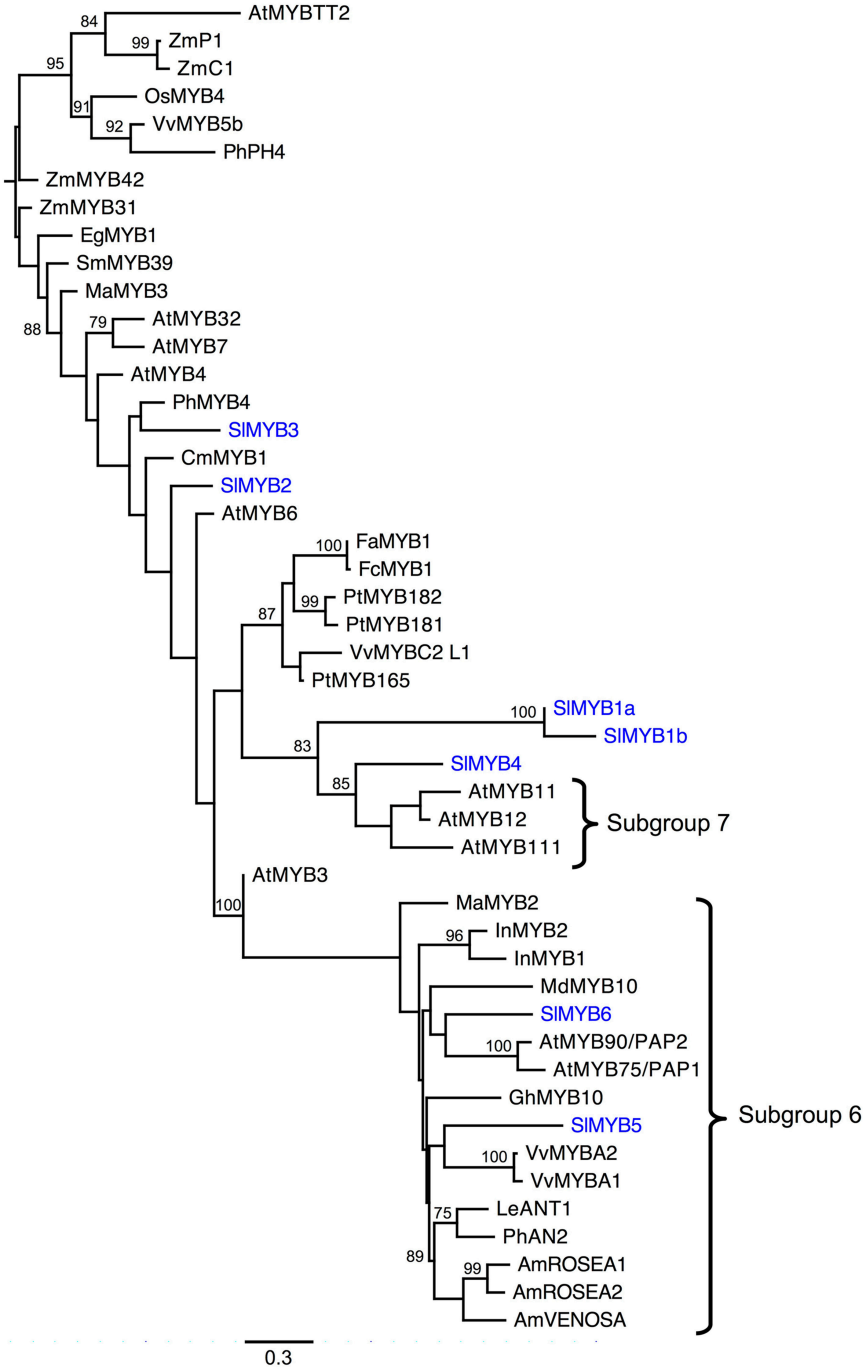
**Phylogenetic analysis of Myb R2R3 DNA binding domains for ***S. littorea*** and other model species**. The R2R3 domains of seven Mybs identified in the *S. littorea* petal tanscriptome were aligned and analyzed under maximum likelihood phylogenetic methods. Bootstrap values greater than 70% are indicated at the nodes. Branches are drawn proportional to the number of substitions per site (see scale bar). Species abbreviations: Am, *Antirrhinum majus*; At, *Arabidopsis thaliana*; Cm, *Chrysanthemum morinifolium*; Eg, *Eucaliptus gunnii*; Fa, *Fragaria ananassa*; Fc, *Fragaria chiloensis*; Gh, *Gerbera hybrida*; In, *Ipomoea nil*; Le, *Lycopersicon esculentum*; Ma, *Mimulus aurantiacus*; Md, *Malus domestica*; Sm, *Salvia miltiorrhiza*; Os, *Oryza sativa;* Ph, *Petunia hybrida*; Pt, *Populus trichocarpa*; Sl, *Silene littorea*; Vv, *Vitis vinifera;* Zm, *Zea mays*. Genbank accession numbers can be found in Table S1 in Supplementary Material.

The phylogenetic analysis of the R2R3 *Myb* DNA binding domain including several ABP-related *Myb*s from model species indicates numerous *S. littorea Myb*s are likely ABP regulators. RAxML and MrBayes phylogenetic analyses produced nearly identical topologies. For simplicity, we present the RAxML results (Figure 3). In particular, *SlMyb4, SlMyb1a*, and *SlMyb1b*, are strongly supported as sister to Subgroup 7, which controls the first dedicated steps of the ABP gene regulation including *F3h* in *A. thaliana* (Stracke et al., 2007; Dubos et al., 2010). *SlMyb5* and *SlMyb6* grouped with a large number of unresolved eudicot *Mybs* of Subgroup 6 which are known to control the later genes of the ABP (Figure 3; Dubos et al., 2010). Both of these mybs have the expected bHLH interaction residues and the ANDV motif in the R3 domain that are characteristic of Subgroup 6 (results not shown). *SlMyb2* and *SlMyb3* are less likely involved in the blockage of the ABP since they are not closely related to exemplars from Subgroups 6 or 7. Both of these mybs have the bHLH binding domain and the C1 conserved motif, but the C2 motif is only present in *SlMyb3* (Dubos et al., 2010; Yoshida et al., 2015).

### Identification and quantification of flavonoids

**Figure 4:**
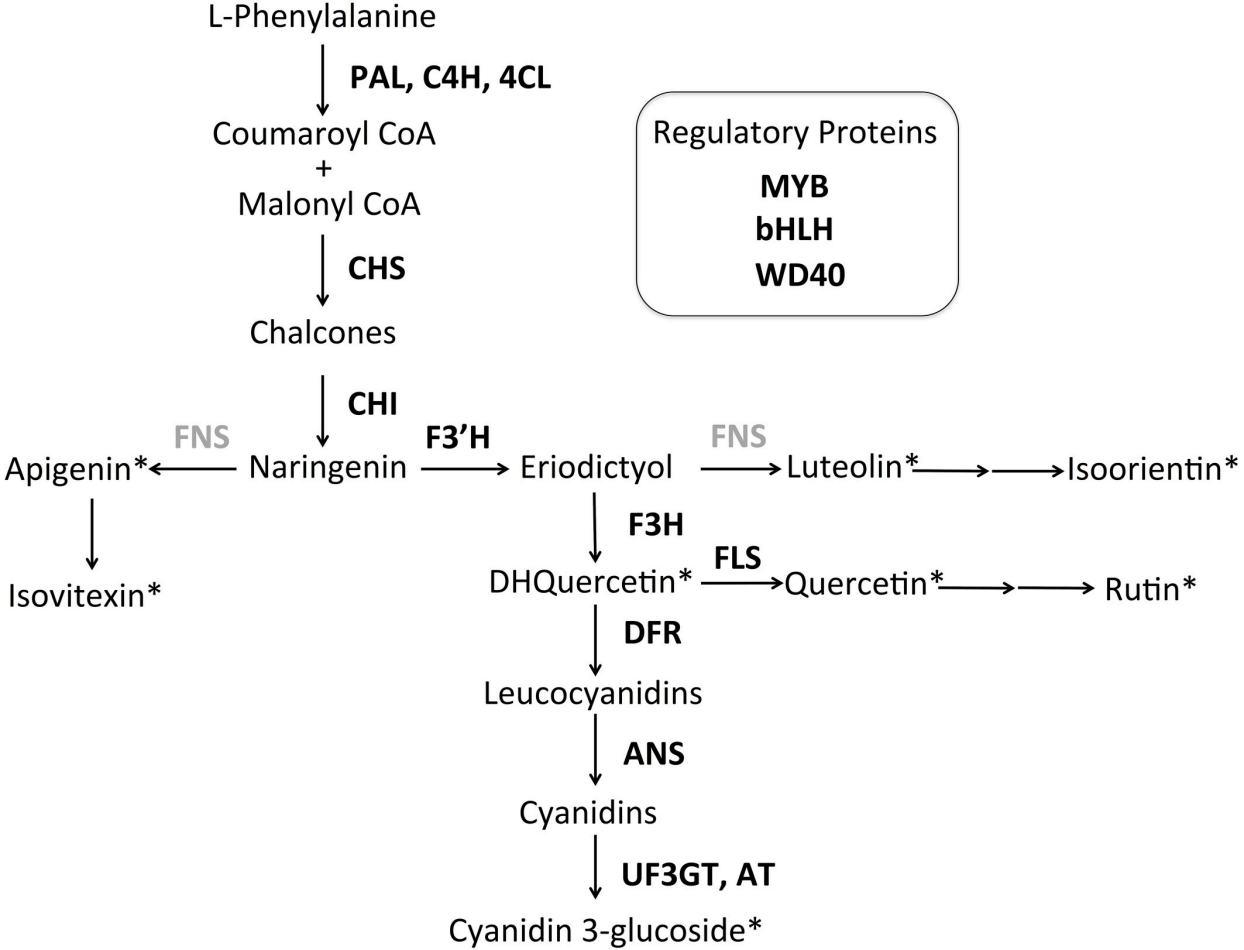
**Tentative anthocyanin biosynthetic pathway for ***S. littorea*****. The pathway is primarily linear with three side branches that produce the anthocyanin pigment cyanidin. Flavonoids detected by HPLC are indicated with asterisks. Enzymes not included in this study are indicated in gray. Enzyme abbreviations are indicated next to arrows: PAL, phenylalanil ammonia-lyase; C4H, cinnamate 4-hydroxylase; 4CL, coumarate CoA ligase; CHS: chalcone synthase; CHI, chalcone isomerase; F3′H, flavonoid 3′hydroxylase; FNS, flavone synthase; F3H, flavanone-3-hydroxylase; FLS, flavonol synthase; DFR, dihydroflavonol 4-reductase; ANS, anthocyanidin synthase; UF3GT, flavonoid-3-O-glucosyltransferase; AT, acyltransferase. The three gene regulatory complex consists of a basic Helix Loop Helix protein (bHLH), WD Repeats (WD40) and R2R3-MYB domains (MYB) in most angiosperms.

HPLC analysis revealed three anthocyanin compounds (glycosylated cyanidin derivatives) responsible for the petal color in *S. littorea* (Table S5 in Supplementary Material). We detected seven additional flavonoids: four flavones (identified from standards as apigenin, isoorientin, isovitexin and luteolin), two flavonols (quercetin and rutin), and one dihydroflavonol (dihydroquercetin). No flavonoids matching the flavanone narigenin nor the isoflavone genisteine, from the earliest dedicated steps of the ABP, were detected. The putative location of these flavonoid intermediates in the ABP is shown in Figure 4.

**Figure 5:**
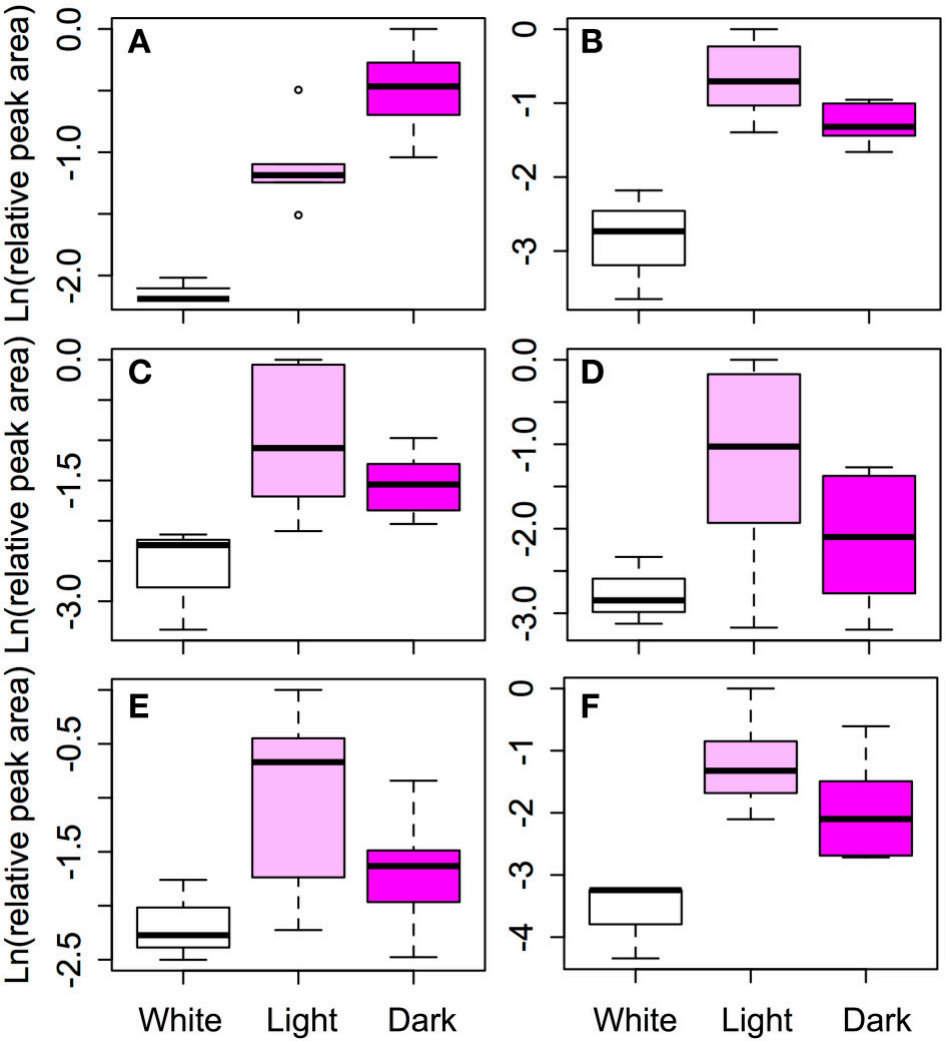
**Comparison of flavonoid concentrations among color morphs**. Six classes of flavonoids were identified by HPLC: **(A)** Cyanidin, **(B)** Rutin, **(C)** Quercetin, **(D)** Luteolin, **(E)** Apigenin, and **(F)** Isovitexin. Cyanidin is the primary anthocyanin pigment. The remaining five flavonoids are intermediates in the pathway (see Figure 4). Log transformed, size corrected relative peak areas are compared for white, light pink, and dark pink samples. The boxes represent the 25th and 75th percentiles, the whiskers are the 5th and 95th percentiles, the central solid lines are the median values, and circles represents outliers.

We compared the relative amounts of anthocyanins and their intermediates across color morphs to link the transcriptome results to the phenotype. The amount of cyanidin derivatives significantly increased with the intensity of the petal color as expected (Table 4, Figure 5). In three of the five flavonoid intermediates (rutin, isovitexin and quercetin), the relative amounts of metabolites in the color morphs were significantly different. Amounts of luteolin derivatives and apigenin were not significantly different among the color morphs (Table 4, Figure 5). *Post-hoc* pairwise comparisons among the three color morphs indicate that differences were always strongest between pigmented and white petals, except for quercetin where white and pink were not significantly different from each other. Light and dark pigmented morphs did not differ in the relative amount of any of the five flavonoid intermediates (Table 4, Figure 5).

**Table 4:** **Statistical analysis of petal flavonoid concentrations. ANOVA results and pairwise Tukey *post-hoc* analyses for significant differences of flavonoid concentrations among the three color morphs (dark pink, light pink, and white). Tukey *post-hoc* tests were only performed on flavonoids with significant ANOVA. Significant Tukey *post-hoc* results (*p* < 0.05) are indicated in bold**.

**Flavonoid**	**ANOVA F-statistics**	**Pairwise Tukey** ***post-hoc p*****-values**		
		**White—Light**	**White—Dark**	**Light—Dark**
Cyanidin^a^	14.18^***^	**0.046**	**0.001**	**0.024**
Rutin	19.71^***^	**0.001**	**0.002**	0.120
Quercetin	5.56^*^	**0.015**	0.108	0.390
Luteolin^b^	3.10	–	–	–
Apigenin	3.72	–	–	–
Isovitexin	10.25^**^	**0.002**	**0.022**	0.243

a *The three cyanidin derivatives were pooled in the ANOVA because our MS/MS quantification cannot differentiate among them*.

b *Luteolin and isoorientin (luteolin hexoside) were pooled for the same reason*.

* *P < 0.05*;

** *P < 0.01*;

*** *P < 0.001*.

## Discussion

We sequenced and measured expression of all ABP-related genes from the petals of the non-model species, *S. littorea*. We assembled complete coding sequences of 28 out of 29 ABP-related loci and identified over 600 SNPs, yet none are sufficient to confer a structural blockage in the ABP. This study is the first to sequence and measure expression of structural and regulatory genes of the ABP in the petals of *Silene*. A previous transcriptome analysis of white flowered *S. vulgaris* has been completed, but it utilized pooled RNA from leaves, roots and whole flowers (Blavet et al., 2011; Sloan et al., 2012). Recently, RNA-Seq studies in non-model species of *Mimulus, Muscari*, and *Parrya* have similarly used *de novo* assemblies followed by expression analyses of the ABP genes (Butler et al., 2014; Lou et al., 2014; Yuan et al., 2014). As in *Silene littorea*, these studies also identified the ABP genes and some of the regulatory loci, confirming our result that RNA-Seq is an efficient tool for narrowing the number of candidate genes responsible for a flower color polymorphism. We assembled the complete CDS of most of the ABP loci (28 out of 29) compared to an average of 71% in *Muscari* and 89% in *Parrya* likely due to the excessive coverage following the replicate sequencing runs (Butler et al., 2014; Lou et al., 2014).

The concentration of synonymous SNPs that correlated with flower color in *Ans* was initially encouraging and warranted further investigation. Unfortunately, none of these SNPs consistently differentiate the flower color samples (highest D-W allele frequency difference is 0.45). Given that there are numerous non-color differentiating SNPs on either side of the cluster of color-related SNPs, it is unlikely that we surveyed a region adjacent to a structural blockage in the ABP at ANS. Furthermore, since none of these SNPs cause any changes to the amino acids, they should have no structural effect on the enzymes activity. Lastly, these SNPs are unlikely to cause regulatory changes since in *Arabidopsis* and *Ipomoea*, the regulation of *Ans* occurs in the promoter (Dong et al., 2014; Xu et al., 2014) where MYB and bHLH binding sites are found.

The expression analysis identified several significantly differentially expressed genes in the petal buds where there was substantially lower expression in white samples compared to pigmented samples. Although we have focused our interpretation of expression differences on the developmental stage closest to when the pigment difference between pink and white become apparent (bud), we have provided results for later petal developmental stages as well (Table S4 in Supplementary Material). In particular, *F3h1* exhibits significantly different expression for both dark pink vs. white and light pink vs. white comparisons. In fact, these are the two largest fold-changes in expression of pigmented vs. white among all ABP-related loci. Changes in *F3h1* expression could be due to mutations in the *cis*-regulatory elements of the promoter or changes in the *trans*-acting myb-bHLH-WD40 regulatory complex. Unfortunately, there is no DNA sequence variation in the 129 bp of the 5′ UTR that we have sequenced, thereby limiting our ability to associate this region with any adjacent *cis*-acting color-differentiating SNPs. Mutations upstream from the 5′ UTR cannot be excluded as a cause of *F3h1* downregulation in *S. littorea* since this locus can affect flower color as has been reported in other species (Dedio et al., 1995; van Houwelingen et al., 1998). Nishihara et al. (2014) found that a white-petaled *Torenia* was caused by a retrotransposon in the promoter of the *F3h* gene. In addition, antisense suppression of *F3h* in carnation resulted in a variety of transgenic plants showing a range of loss of function, from subtle attenuation to complete loss (Zuker et al., 2002).

The regulation of ABP genes through changes in expression of their regulatory elements, could also lead to the differential expression observed in *F3h*. In *Mimulus aurantiacus, MaMyb2* regulates the expression of *F3h, Dfr*, and *Ans*. When *MaMyyb2* was silenced, the expression of these genes was significantly lower than the control (Streisfeld et al., 2013). In *S. littorea*, the gene tree of MYBs, placed *SlMyb5* and *SlMyb6* in the same group as *MaMyb2* and many other known ABP regulators (Subgroup 7 according to Dubos et al., 2010), and also presented a reduction of expression in the non-anthocyanin morph, although not significant. This result highlights *SlMyb5* and *SlMyb6* as tentative regulators of the expression of the ABP genes, however further experiments are needed to test this hypothesis.

Significant differences in expression were also found in *C4h2* (dark pink vs. white), *F3*′*h* (light pink vs. white) and the transcription factor *Myb1a* (dark pink vs. white and dark pink vs. light pink). *C4h2* is a pre-ABP gene acting in the general phenylpropanoid biosynthetic pathway (Ehlting et al., 2006). Since we did not detect the biochemical product of C4H (nor were the products of CHS and CHI, chalcone and naringinen, identified), we cannot differentiate whether there is a blockage at C4H due to decreased expression or if the enzymes downstream of C4H are consuming all of the product during flux down the ABP. Interestingly, suppression of the first two dedicated genes of ABP such as *Chs* or *Chi* would eliminate most flavonoid intermediates without affecting the production of upstream compounds including the volatile benzenoids responsible for floral scent (Clark and Verwoerd, 2011). This is because *C4h2, Chs*, and *Chi* are all located downstream of the production of cinnamic acid, the initial substrate of this side branch (Davies and Schwinn, 2006; Ben Zvi et al., 2008). Although differences were not significant, expression of *C4h2* was also much higher in light vs. white morphs. The phylogenetic tree of *Mybs* showed that *SlMyb1a* (closely related to *SlMyb1b*, with 68% of amino acid similarity) and *SlMyb4* are closely related to Subgroup 7 (*sensu* Dubos et al., 2010) which controls several genes in the first half of the ABP including *F3h* in *A. thaliana* (Dubos et al., 2010). On the other hand, we also found that light pink and dark pink petals showed differential expression in two *Myb* transcriptional regulators. This suggests that differences in color intensity between the light and dark pink morphs could be due to a different candidate ABP locus than the loss-of-function (e.g., Hopkins and Rausher, 2011; Yuan et al., 2013), rather than heterozygosity of a single loss-of-function locus in the white morph. In fact, during the SNP assignment, the light pink morph never had a higher probability of being heterozygote, however confirming the number of loci responsible for these three color morphs must be evaluated with an F_2_ population that is segregating for flower color.

A structural or regulatory blockage in the ABP would decrease the amount of flavonoid intermediates below the blockage, but would increase the amount of intermediates in upstream side branches (depending on the dynamics of metabolite flux through the pathway). The flavonoid biochemical analysis identified cyanidin as the primary anthocyanin and an additional five flavonoid intermediates to compare among the color morphs. Only three of them were significantly different between white and pigmented individuals and two (quercetin and rutin) are consistent with a blockage at or above F3H. Consistent results between HPLC and expression analysis were found in *Parrya nudicaulis*, where the white morph did not produce catechins or flavonols due to the reduced expression in *Chs* (Dick et al., 2011). Nevertheless, in *Iris lutescens*, the production of non-anthocyanic flavonoids (including chalcones, flavones and flavonols) in the yellow morph was higher than in the purple (Wang et al., 2013). In *Muscari armeniacum*, Lou et al. (2014) found that except for anthocyanins (delphinidin and cyanidin), the white morph contained the same metabolites as the blue, and generally at higher concentrations. They argue that the blockage in DFR in the white morph, caused a redirection of the flux of metabolites through a side-chain to other products. A similar argument may hold between the light and pink morphs in *S. littorea*. Although differences were not significant, the light morph of *S. littorea* showed a trend toward higher concentrations of flavonols and flavones compared with the dark pink morph (Figure 5), which could be due to a redirection of the flux of metabolites.

Based on our biochemical analysis, we have proposed a tentative metabolic pathway of anthocyanin in the petals of *S. littorea* (Figure 4). The pink color of the petals is caused by the accumulation of cyanidin 3-glucoside derivatives, as is found in *S. armeria* (Iwashina and Ootani, 1987) and *S. dioica* (Kamsteeg et al., 1979). Dark pink flowers in *S. littorea* showed the same cyanidin 3-glucoside derivatives but in a much higher concentration than light pink flowers, which suggest that the pink intensity is caused by the different concentration of these compounds. In other species, it has been proposed that co-pigments such as flavones and flavonols play an important role in the color or intensity of the petals (Gould and Lister, 2006; Thill et al., 2012; Nishihara et al., 2014). For example, brown color of outer part of the labellum of *Ophrys speculum* is suggested to be caused by the flavonols acting as co-pigments of cyanidins (Vignolini et al., 2012). In *S. littorea*, flavonols and flavones are not expected to play a key role in the pink intensity since higher concentrations were not found in darker petals.

The lack of anthocyanins, and the lower levels of other flavonoids in petals of the white-flowered morph of *S. littorea* could result in a fitness disadvantage in stressful conditions. These pigments (and some of their intermediates) are known to influence pollinator visitation, attraction of florivores and susceptibility to pathogens (e.g., Hoballah et al., 2007; Johnson et al., 2008; Falcone Ferreyra et al., 2012). Furthermore, anthocyanin-less morphs may more susceptible to abiotic stresses such as heat, cold and dessication (reviewed in Winkel-Shirley, 2002). Interestingly, individuals of the white campion, *S. pratensis*, lacking glyscosilated isovitexin showed ruptured upper epidermal cells that caused curved petals (Van Brederode et al., 1982). The possible disadvantage of the lack of anthocyanins or other flavonoids can be even higher when vegetative tissues are also affected (Levin and Brack, 1995; Warren and Mackenzie, 2001). This could be the case of a different type of white-flowered mutant that appears rarely in a few southern populations of *S. littorea*. This whole-plant mutant is not able to produce anthocyanins in other tissues of the plant (see the calyx in Figure S1E in Supplementary Material), and is found at very low frequencies (<0.05%; Casimiro-Soriguer, 2015). Mutations in structural genes are commonly responsible for low frequency white-flowered mutants in several other species (i.e., Coberly and Rausher, 2008; Wu et al., 2013). Thus, the rare mutant in *S. littorea* could be also due to a coding mutation, but future experiments should be carried out to answer this question. However, the high frequency white-flowered mutant studied here, is able to produce anthocyanins and flavonoids in other tissues of the plant including calyx, leaves and stem (see calyx in Figure S1E in Supplementary Material). Instead, we posit that regulatory changes in *SlMyb1a* affects expression of *SlF3h1* (at least) which is then the most likely blockage in the ABP for these northwestern Iberian white-flowered morphs of *S. littorea*.

## Conclusions

We used RNA-Seq to simultaneously sequence and estimate expression of 29 ABP-related loci among three flower color morphs of the non-model plant, *S. littorea*. After sequencing the complete coding regions of all structural genes and most regulatory loci, we found a cluster of nine synonymous SNPs around the intron in *Ans* whose frequencies differ among color morphs, yet their functional significance is unclear. Additional sampling confirmed these *Ans* allele frequency differences, yet no single SNP consistently differentiates the color morphs. Instead, there is consistent and significant downregulation in the expression of *F3h* when comparing pigmented and white petal buds which may be influenced by decreased expression of *Myb1a*—a regulator of *F3h* in other eudicots. The flavonoid biochemical analysis is partially consistent with downregulation of *F3h*—the most likely blockage in the ABP leading to the loss of floral anthocyanins potentially mediated by expression of *Myb1a*. Expanded sampling of white and dark individuals for expression analysis of *SlMyb1a* and *F3h* and sequencing of the promoter region in association with genetic analysis of these loci using a segregating F_2_ population are essential steps to validating these results.

## Author contributions

JW, MB, EN, and IS conceived and designed the experiments. MB, JD, EN, and IS carried out the sampling. JW and IS performed the assembly and the sequences comparison. JW and JD performed the Sanger sequencing. JD, JW, and IS run the phylogenetic analysis. MB and IS carried out the differential expression analysis. EN, JD, and IS analyzed the HPLC data. JW, EN, MB, and IS drafted the manuscript. All authors read and approved the final manuscript.

### Conflict of interest statement

The authors declare that the research was conducted in the absence of any commercial or financial relationships that could be construed as a potential conflict of interest.

## References

[B1] Albert N. W. Davies K. M. Lewis D. H. Zhang H. Montefiori M. Brendolise C.. (2014). A conserved network of transcriptional activators and repressors regulates anthocyanin pigmentation in eudicots. Plant Cell 26, 962–980. 10.1105/tpc.113.122069 24642943 PMC4001404

[B2] Anders S. Huber W. (2010). Differential expression analysis for sequence count data. Genome Biol. 11:R106. 10.1186/gb-2010-11-10-r106 20979621 PMC3218662

[B3] Ben Zvi M. M. Negre Zakharov F. Masci T. Ovadis M. Shklarman E. Ben Meir H.. (2008). Interlinking showy traits: co engineering of scent and colour biosynthesis in flowers. Plant Biotechnol. J. 6, 403–415. 10.1111/j.1467-7652.2008.00329.x 18346094

[B4] Bernasconi G. Antonovics J. Biere A. Charlesworth D. Delph L. F. Filatov D.. (2009). *Silene* as a model system in ecology and evolution. Heredity 103, 5–14. 10.1038/hdy.2009.34 19367316

[B5] Blavet N. Charif D. Oger-Desfeux C. Marais G. A. B. Widmer A. (2011). Comparative high-throughput transcriptome sequencing and development of SiESTa, the *Silene* EST annotation database. BMC Genomics 12:376. 10.1186/1471-2164-12-376 21791039 PMC3157477

[B6] Bradshaw H. D. Wilbert S. M. Otto K. G. Schemske D. W. (1995). Genetic mapping of floral traits associated with reproductive isolation in monkeyflowers (*Mimulus*). Nature 376, 762–765. 10.1038/376762a0

[B7] Brockington S. F. Walker R. H. Glover B. J. Soltis P. S. Soltis D. E. (2011). Complex pigment evolution in the Caryophyllales. New Phytol. 190, 854–864. 10.1111/j.1469-8137.2011.03687.x 21714182

[B8] Butler T. Dick C. Carlson M. L. Whittall J. B (2014). Transcriptome analysis of a petal anthocyanin polymorphism in the arctic mustard, *Parrya nudicaulis*. PLoS ONE 9:e101338. 10.1371/journal.pone.0101338 25033465 PMC4102464

[B9] Campanella J. J. Smalley J. V. Dempsey M. E. (2014). A phylogenetic examination of the primary anthocyanin production pathway of the Plantae. Bot. Stud. 55:10 10.1186/1999-3110-55-10 PMC5432750 28510914

[B10] Casimiro-Soriguer I. (2015). Sistemas Sexuales y Polimorfismo de Color en Silene: una Aproximación en la Sección Psammophilae. Ph.D. dissertation. Seville: Pablo de Olavide University.

[B11] Casimiro-Soriguer I. Buide M. L. Narbona E. (2013). The roles of female and hermaphroditic flowers in the gynodioecious-gynomonoecious *Silene littorea*, insights into the phenology of sex expression. Plant Biol. 15, 941–947. 10.1111/j.1438-8677.2012.00697.x 23174011

[B12] Clark S. T. Verwoerd W. S. (2011). A systems approach to identifying correlated gene targets for the loss of colour pigmentation in plants. BMC Bioinformatics 12:343. 10.1186/1471-2105-12-343 21849042 PMC3180701

[B13] Coberly L. C. Rausher M. D. (2008). Pleiotropic effects of an allele producing white flowers in *Ipomoea purpurea*. Evolution 62, 1076–1085. 10.1111/j.1558-5646.2008.00355.x 18298642

[B14] Dardanelli M. S. Fernández de Córdoba F. J. Espuny M. R. Rodríguez Carvajal M. A. Soria Díaz M. E. Gil Serrano A. M. (2008). Effect of *Azospirillum brasilense* coinoculated with *Rhizobium* on *Phaseolus vulgaris* flavonoids and Nod factor production under salt stress. Soil Biol. Biochem. 11, 2713–2721. 10.1016/j.soilbio.2008.06.016

[B15] Davies K. M. (2009). Modifying anthocyanin production in flowers, in Anthocyanins: Biosynthesis, Functions, and Applications, eds Gould K. Davies K. M. Winefield C. (New York, NY: Springer), 49–83.

[B16] Davies K. M. Schwinn K. E. (2006). Molecular biology and biotechnology of flavonoid biosynthesis, in Flavonoids: Chemistry, Biochemistry and Applications, eds Andersen Ø. M. Markham K. R. (Boca Raton, FL: CRC Press), 143–218.

[B17] Dedio J. Saedler H. Forkmann G. (1995). Molecular cloning of the flavanone 3ß-hydroxylase gne (FHT) from carnation (*Dianthus caryophyllus*) and analysis of stable and unstable FHT mutants. Theor. Appl. Genet. 90, 611–617. 10.1007/BF00222123 24174017

[B18] Del Valle J. C. Buide M. L. Casimiro-Soriguer I. Whittall J. B. Narbona E. (2015). On flavonoid accumulation in different plant parts: variation patterns among individuals and populations in the shore campion (*Silene littorea*). Front. Plant Sci. 6:939. 10.3389/fpls.2015.00939 26579180 PMC4625047

[B19] DePristo M. Banks E. Poplin R. Garimella K. Maguire J. Hartl C.. (2011). A framework for variation discovery and genotyping using next-generation DNA sequencing data. Nat. Genet. 43, 491–498. 10.1038/ng.806 21478889 PMC3083463

[B20] Dick C. A. Buenrostro J. Butler T. Carlson M. L. Kliebenstein D. J. Whittall J. B. (2011). Arctic Mustard flower color polymorphism controlled by petal-specific downregulation at the threshold of the anthocyanin biosynthetic pathway. PLoS ONE 6:e18230. 10.1371/journal.pone.0018230 21490971 PMC3072389

[B21] Dong W. You Y. Niu L. Gao F. (2014). Isolation and analysis of the promoter of an anthocyanin synthase gene from purple-fleshed sweet potato tubers. Acta Physiol. Plant. 36, 2637–2649. 10.1007/s11738-014-1635-4

[B22] Dubos C. Stracke R. Grotewold E. Weisshaar B. Martin C. Lepiniec L. (2010). MYB transcription factors in *Arabidopsis*. Trends Plant Sci. 10, 573–581. 10.1016/j.tplants.2010.06.005 20674465

[B23] Eckhart V. M. Rushing N. S. Hart G. M. Hansen J. D. (2006). Frequency-dependent pollinator foraging in polymorphic *Clarkia xantiana* ssp. xantiana populations: implications for flower colour evolution and pollinator interactions. Oikos 112, 412–421. 10.1111/j.0030-1299.2006.14289.x

[B24] Ehlting J. Hamberger B. Million-Rousseau R. Werck-Reichhart D. (2006). Cytochromes P450 in phenolic metabolism. Phytochem. Rev. 5, 239–270. 10.1007/s11101-006-9025-1

[B25] Ellis T. H. Hofer J. M. Timmerman-Vaughan G. M. Coyne C. J. Hellens R. P. (2011). Mendel, 150 years on. Trends Plant Sci. 16, 590–596. 10.1016/j.tplants.2011.06.006 21775188

[B26] Falcone Ferreyra M. L. Rius S. P. Casati P. (2012). Flavonoids: biosynthesis, biological functions, and biotechnological applications. Front. Plant Sci. 3:222. 10.3389/fpls.2012.00222 23060891 PMC3460232

[B27] Fossen T. Andersen Ø. M. (2006). Spectroscopic techniques applied to flavonoids, in Flavonoids: Chemistry, Biochemistry and Applications, eds Andersen Ø. M. Markham K. R. (Boca Raton, FL: CRC Press), 37–142.

[B28] Gould K. S. Lister C. (2006). Flavonoid functions in plants, in Flavonoids: Chemistry, Biochemistry and Applications, eds Andersen Ø. M. Markham K. R. (Boca Raton, FL: CRC Press), 397–442.

[B29] Grotewold E. (2006). The genetics and biochemistry of floral pigments. Annu. Rev. Plant Biol. 57, 761–780. 10.1146/annurev.arplant.57.032905.105248 16669781

[B30] Hall T. A. (1999). BioEdit: a user-friendly biological sequence alignment editor and analysis program for Windows 95/98/NT. Nucleic Acids Symp. Ser. 41, 95–98.

[B31] Hichri I. Barrieu F. Bogs J. Kappel C. Delrot S. Lauvergeat V. (2011). Recent advances in the transcriptional regulation of the flavonoid biosynthetic pathway. J. Exp. Bot. 62, 2465–2483. 10.1093/jxb/erq442 21278228

[B32] Hoballah M. E. Gübitz T. Stuurman J. Broger L. Barone M. Mandel T.. (2007). Single-gene-mediated shift in pollinator attraction in *Petunia*. Plant Cell 19, 779–790. 10.1105/tpc.106.048694 17337627 PMC1867374

[B33] Holton T. A. Cornish E. C. (1995). Genetics and biochemistry of anthocyanin biosynthesis. Plant Cell 7, 1071–1083. 10.1105/tpc.7.7.1071 12242398 PMC160913

[B34] Hopkins R. Rausher M. D. (2011). Identification of two genes causing reinforcement in the Texas wildflower *Phlox drummondii*. Nature 469, 411–414. 10.1038/nature09641 21217687

[B35] Huelsenbeck J. P. Ronquist F. (2001). MRBAYES: Bayesian inference of phylogenetic trees. Bioinformatics 17, 754–755. 10.1093/bioinformatics/17.8.754 11524383

[B36] Irwin R. E. Strauss S. Y. (2005). Flower color microevolution in wild radish: evolutionary response to pollinator-mediated selection. Am. Nat. 165, 225–237. 10.1086/426714 15729652

[B37] Iwashina T. Ootani S. (1987). Characterization of C-glycosylflavones and anthocyanins in several species of Caryophyllaceae. Ann. Tsukuba Bot. Gard. 20, 19–30.

[B38] Johnson E. T. Berhow M. A. Dowd P. F. (2008). Colored and white sectors from star-patterned *Petunia* flowers display differential resistance to corn earworm and cabbage looper larvae. J. Chem. Ecol. 34, 757–765. 10.1007/s10886-008-9444-0 18484139

[B39] Kamsteeg J. van Brederode J. van Nigtevecht G. (1979). Genetics of anthocyanin formation in petals of the red campion (*Silene dioica* (L.) Clairv). Genetica 51, 5–13. 10.1007/BF00139492

[B40] Kopp A. (2009). Metamodels and phylogenetic replication: a systematic approach to the evolution of developmental pathways. Evolution 63, 2771–2789. 10.1111/j.1558-5646.2009.00761.x 19545263

[B41] Lee W.-P. Stromberg M. P. Ward A. Stewart C. Garrison E. P. Marth G. T. (2014). MOSAIK: a hash-based algorithm for accurate next-generation sequencing short-read mapping. PLoS ONE 9:e90581. 10.1371/journal.pone.0090581 24599324 PMC3944147

[B42] Levin D. A. Brack E. T. (1995). Natural selection against white petals in *Phlox*. Evolution 49, 1017–1022. 10.2307/2410423 28564870

[B43] Li X. Sun H. Pei J. Dong Y. Wang F. Chen H.. (2012). *De novo* sequencing and comparative analysis of the blueberry transcriptome to discover putative genes related to antioxidants. Gene 511, 54–61. 10.1016/j.gene.2012.09.021 22995346

[B44] Li X. Xiong Z. Ying X. Cui L. Zhu W. Li F. (2006). A rapid ultra-performance liquid chromatography-electrospray ionization tandem mass spectrometric method for the qualitative and quantitative analysis of the constituents of the flower of *Trollius ledibouri* Reichb. Anal. Chim. Acta 580, 170–180. 10.1016/j.aca.2006.07.069 17723770

[B45] Lou Q. Liu Y. Qi Y. Jiao S. Tian F. Jiang L.. (2014). Transcriptome sequencing and metabolite analysis reveals the role of delphinidin metabolism in flower color in grape hyacinth. J. Exp. Bot. 65, 3157–3164. 10.1093/jxb/eru168 24790110 PMC4071837

[B46] Lulin H. Xiao Y. Pei S. Wen T. Shangqin H. (2012). The first Illumina-based *de novo* transcriptome sequencing and analysis of safflower flowers. PLoS ONE 7:e38653. 10.1371/journal.pone.0038653 22723874 PMC3378585

[B47] Martins T. R. Berg J. J. Blinka S. Rausher M. D. Baum D. A. (2013). Precise spatio-temporal regulation of the anthocyanin biosynthetic pathway leads to petal spot formation in *Clarkia gracilis* (Onagraceae). New Phytol. 197, 958–969. 10.1111/nph.12062 23231386 PMC3540125

[B48] McKenna A. Hanna M. Banks E. Sivachenko A. Cibulskis K. Kernytsky A.. (2010). The Genome Analysis Toolkit: a MapReduce framework for analyzing next-generation DNA sequencing data. Genome Res. 20, 1297–1303. 10.1101/gr.107524.110 20644199 PMC2928508

[B49] Mendel G. (1866). Versuche über Pflanzen-Hybriden. Verhandlungen des Naturforschenden Vereines Brünn. English translation, Available online at: http://www.mendelweb.org/MWarchive.html

[B50] Miller R. Owens S. J. Rørslett B. (2011). Plants and color: flowers and pollination. Opt. Laser Technol. 43, 282–294. 10.1016/j.optlastec.2008.12.018

[B51] Napoli C. Lemieux C. Jorgensen R. (1990). Introduction of a chimeric chalcone synthase gene into petunia results in reversible co-suppression of homologous genes in trans. Plant Cell 2, 279–289. 10.1105/tpc.2.4.279 12354959 PMC159885

[B52] Nishihara M. Yamada E. Saito M. Fujita K. Takahashi H. Nakatsuka T. (2014). Molecular characterization of mutations in white-flowered torenia plants. BMC Plant Biol. 14:86. 10.1186/1471-2229-14-86 24694353 PMC4234012

[B53] Oxelman B. Rautenberg A. Thollesson M. Larsson A. Frajman B. Eggens F. (2013). Sileneae Taxonomy and Systematics. Available online at: http://www.Sileneae.info

[B54] Qiao S. Shi R. Liu M. Zhang C. Yang W. Shi X.. (2011). Simultaneous quantification of flavonoids and phenolic acids in Herba Scutellariae barbatae and its confused plants by high performance liquid chromatography-tandem mass spectrometry. Food Chem. 129, 1297–1304. 10.1016/j.foodchem.2011.05.064 25212370

[B55] R Core Team (2013). R: A Language and Environment for Statistical Computing. Available online at: http://www.R-project.org/

[B56] Rutherford K. Parkhill J. Crook J. Horsnell T. Rice P. Rajandream M. A.. (2000). Artemis: sequence visualization and annotation. Bioinformatics 16, 944–945. 10.1093/bioinformatics/16.10.944 11120685

[B57] Schemske D. W. Bierzychudek P. (2001). Perspective: evolution of flower color in the desert annual *Linanthus parryae*: wright revisited. Evolution 55, 1269–1282. 10.1111/j.0014-3820.2001.tb00650.x 11525452

[B58] Schulz M. H. Zerbino D. R. Vingron M. Birney E. (2012). Oases: robust *de novo* RNA-seq assembly across the dynamic range of expression levels. Bioinformatics 28, 1086–1092. 10.1093/bioinformatics/bts094 22368243 PMC3324515

[B59] Schwinn K. Venail J. Shang Y. Mackay S. Alm V. Butelli E.. (2006). A small family of MYB-regulatory genes controls floral pigmentation intensity and patterning in the genus *Antirrhinum*. Plant Cell 18, 831–851. 10.1105/tpc.105.039255 16531495 PMC1425845

[B60] Sloan D. B. Keller S. R. Berardi A. E. Sanderson B. J. Karpovich J. F. Taylor D. R. (2012). *De novo* transcriptome assembly and polymorphism detection in the flowering plant *Silene vulgaris* (Caryophyllaceae). Mol. Ecol. Resour. 12, 333–343. 10.1111/j.1755-0998.2011.03079.x 21999839

[B61] Sobel J. M. Streisfeld M. A. (2013). Flower color as a model system for studies of plant evo-devo. Front. Plant Sci. 4:321. 10.3389/fpls.2013.00321 23970892 PMC3748380

[B62] Stamatakis A. (2014). RAxML version 8: a tool for phylogenetic analysis and post-analysis of large phylogenies. Bioinformatics 30, 1312–1313. 10.1093/bioinformatics/btu033 24451623 PMC3998144

[B63] Stern D. L. Orgogozo V. (2008). The loci of evolution: how predictable is genetic evolution? Evolution 62, 2155–2177. 10.1111/j.1558-5646.2008.00450.x 18616572 PMC2613234

[B64] Stracke R. Ishihara H. Barsch G. H. A. Mehrtens F. Niehaus K. Weisshaar B. (2007). Differential regulation of closely related R2R3-MYB transcription factors controls flavonol accumulation in different parts of the *Arabidopsis thaliana* seedling. Plant J. 50, 660–677. 10.1111/j.1365-313X.2007.03078.x 17419845 PMC1976380

[B65] Strauss S. Whittall J. B. (2006). Non-pollinator agents of selection on floral traits, in Ecology and Evolution of Flowers, eds Harder L. D. Barrett S. C. H. (Oxford: Oxford University Press), 120–138.

[B66] Streisfeld M. A. Rausher M. D. (2011). Population genetics, pleiotropy, and the preferential fixation of mutations during adaptive evolution. Evolution 65, 629–642. 10.1111/j.1558-5646.2010.01165.x 21054357

[B67] Streisfeld M. A. Young W. N. Sobel J. M. (2013). Divergent selection drives genetic differentiation in an R2R3-MYB transcription factor that contributes to incipient speciation in *Mimulus aurantiacus*. PLoS Genet. 9:e1003385. 10.1371/journal.pgen.1003385 23555295 PMC3605050

[B68] Swarbreck D. Wilks C. Lamesch P. Berardini T. Z. Garcia-Hernandez M. Foerster H.. (2008). The *Arabidopsis* information resource (TAIR): gene structure and function annotation. Nucleic Acids Res. 36, D1009–D1014. 10.1093/nar/gkm965 17986450 PMC2238962

[B69] Talavera S. (1979). Revisión de la sect. *Erectorefractae* Chowdhuri del género *Silene* L. Lagascalia 8, 135–164.

[B70] Tanaka Y. Sasaki N. Ohmiya A. (2008). Biosynthesis of plant pigments: anthocyanins, betalains and carotenoids. Plant J. 54, 733–749. 10.1111/j.1365-313X.2008.03447.x 18476875

[B71] Thill J. Miosic S. Ahmed R. Schlangen K. Muster G. Stich K.. (2012). ‘Le Rouge et le Noir’: a decline in flavone formation correlates with the rare color of black dahlia (*Dahlia variabilis* hort.) flowers. BMC Plant Biol. 12:255. 10.1186/1471-2229-12-225 23176321 PMC3557166

[B72] Van Brederode J. Van Genderen H. H. Berendsen W. (1982). Morphological effects of the flavone isovitexin in a non-glycosylating genotype of *Silene pratensis* (Caryophyllaceae). Experientia 38, 929–931. 10.1007/BF01953658

[B73] van Houwelingen A. Souer E. Spelt K. Kloos D. Mol J. Koes R. (1998). Analysis of flower pigmentation mutants generated by random transposon mutagenesis in *Petunia hybrida*. Plant J. 13, 39–50. 9680963 10.1046/j.1365-313x.1998.00005.x

[B74] Vignolini S. Davey M. P. Bateman R. M. Rudall P. J. Moyroud E. Tratt J.. (2012). The mirror crack'd: both pigment and structure contribute to the glossy blue appearance of the mirror orchid, *Ophrys speculum*. New Phytol. 196, 1038–1047. 10.1111/j.1469-8137.2012.04356.x 23043621

[B75] Wang H. Conchou L. Bessière J. M. Cazals G. Schatz B. Imbert E. (2013). Flower color polymorphism in *Iris lutescens* (Iridaceae): biochemical analyses in light of plant–insect interactions. Phytochemistry 94, 123–134. 10.1016/j.phytochem.2013.05.007 23790644

[B76] Warren J. Mackenzie S. (2001). Why are all color combinations not equally represented as flower-color polymorphisms? New Phytol. 151, 237–241. 10.1046/j.1469-8137.2001.00159.x 33873384

[B77] Wessinger C. A. Rausher M. D. (2012). Lessons from flower colour evolution on targets of selection. J. Exp. Bot. 63, 5741–5749. 10.1093/jxb/ers267 23048126

[B78] Whittall J. B. Voelckel C. Kliebenstein D. J. Hodges S. A. (2006). Convergence, constraint and the role of gene expression during adaptive radiation: floral anthocyanins in *Aquilegia*. Mol. Ecol. 15, 4645–4657. 10.1111/j.1365-294X.2006.03114.x 17107490

[B79] Winkel-Shirley B. (2002). Biosynthesis of flavonoids and effects of stress. Curr. Opin. Plant Biol. 5, 218–223. 10.1016/S1369-5266(02)00256-X 11960739

[B80] Wright S. (1943). An analysis of local variability of flower color in *Linanthus parryae*. Genetics 28, 139–156. 17247075 10.1093/genetics/28.2.139 PMC1209197

[B81] Wu C. A. Streisfeld M. A. Nutter L. I. Cross K. A. (2013). The genetic basis of a rare flower color polymorphism in *Mimulus lewisii* provides insight into the repeatability of evolution. PLoS ONE 8:e81173. 10.1371/journal.pone.0081173 24312531 PMC3849174

[B82] Xu W. Grain D. Bobet S. Le Gourrierec J. Thévenin J. Kelemen Z.. (2014). Complexity and robustness of the flavonoid transcriptional regulatory network revealed by comprehensive analyses of MYB-bHLH-WDR complexes and their targets in *Arabidopsis* seed. New Phytol. 202, 132–144. 10.1111/nph.12620 24299194

[B83] Xu Y. Gao S. Yang Y. Huang M. Cheng L. Wei Q.. (2013). Transcriptome sequencing and whole genome expression profiling of chrysanthemum under dehydration stress. BMC Genomics 14:662. 10.1186/1471-2164-14-662 24074255 PMC3849779

[B84] Yoshida K. Ma D. Constabel P. (2015). The MYB182 protein down-regulates proanthocyanidin and anthocyanin biosynthesis in poplar by repressing both structural and regulatory flavonoid genes. Plant Physiol. 167, 693–710. 10.1104/pp.114.253674 25624398 PMC4348771

[B85] Yuan Y. W. Sagawa J. M. Frost L. Vela J. P. Bradshaw H. D. Jr. (2014). Transcriptional control of floral anthocyanin pigmentation in monkeyflowers (*Mimulus*). New Phytol. 204, 1013–1027. 10.1111/nph.12968 25103615 PMC4221532

[B86] Yuan Y. W. Sagawa J. M. Young R. C. Christensen B. J. Bradshaw H. D. (2013). Genetic dissection of a major anthocyanin QTL contributing to pollinator-mediated reproductive isolation between sister species of *Mimulus*. Genetics 194, 255–263. 10.1534/genetics.112.146852 23335333 PMC3632473

[B87] Zerbino D. Birney D. (2008). Velvet: algorithms for *de novo* short read assembly using De Brujin graphs. Genome Res. 18, 821–829. 10.1101/gr.074492.107 18349386 PMC2336801

[B88] Zhang F. Gonzalez A. Zhao M. Payne C. T. Lloyd A. (2003). A network of redundant bHLH proteins functions in all TTG1-dependent pathways of *Arabidopsis*. Development 130, 4859–4869. 10.1242/dev.00681 12917293

[B89] Zhao D. Tao J. (2015). Recent advances on the development and regulation of flower color in ornamental plants. Front. Plant Sci. 6:261. 10.3389/fpls.2015.00261 25964787 PMC4410614

[B90] Zuker A. Tzfira T. Ben-Meir H. Ovadis M. Shklarman E. Itzhaki H. (2002). Modification of flower color and frangance by antisense supression of the flavanone 3-hydroxylase gene. Mol. Breed. 9, 33–41. 10.1023/A:1019204531262

