# Supplementary material for: Transcriptome and Biochemical Analysis of a Flower Color Polymorphism in Silene littorea (Caryophyllaceae)
Source: Front Plant Sci. 2016 Feb 29;7:204. doi: 10.3389/fpls.2016.00204 (PMC4770042; doi:10.3389/fpls.2016.00204)
Supplement: Supplementary file 1 [file Table1.DOCX]

**Table S1. Accession numbers for R2R3 *Myb* phylogenetic analysis.** The following R2R3 Myb sequences were included in a phylogenetic analysis with those of *S. littorea* to infer which loci may be involved in anthocyanin and flavonoid regulation.

| Taxon | Gene Name | Genbank Accession # |
| --- | --- | --- |
| *Antirrhinum majus* | AmROSEA1  AmROSEA2  AmVENOSA | DQ275529  DQ275530  DQ275531 |
| *Arabidopsis thaliana* | AtMYBTT2  AtMYB4  AtMYB111  AtMYB12  AtMYB11  AtMYB3  AtMYB6  AtMYB7  AtMYB32  AtMYB75/PAP1  AtMYB90/PAP2 | NM_122946  AY519615  NM_124310  NM_130314  NM_116126  NM_102111  NM_117014  NM_127224  NM_119665  NM_104541  NM_105310 |
| *Chrysanthemum morifolium* | CmMYB1 | JF795917 |
| *Eucalyptus gunnii* | EgMYB1 | AJ576024 |
| *Fragaria ananassa* | FaMYB1 | AF401220 |
| *Fragaria chiloensis* | FcMYB1 | GK867222 |
| *Gerbera hybrida* | GhMYB10 | AJ554700 |
| *Ipomoea nil* | InMYB1  InMYB2 | AB232770  AB234211 |
| *Lycopersicon esculentum* | LeANT1 | AY348870 |
| *Malus domestica* | MdMYB10 | AB744002 |
| *Mimulus aurantiacus* | MaMYB3  MaMYB2 | JX661266  JX661264 |
| *Salvia miltiorrhiza* | SmMYB39 | KC213793 |
| *Oryza sativa* | OsMYB4 | D88620 |
| *Petunia hybrida* | PhPH4  PhAN2  PhMYB4 | AY973324  AF146702  HM447143 |
| *Populus trichocarpa* | PtMYB165  PtMYB181  PtMYB182 | XM_002315854  XM_002301658  XM_002305836 |
| *Vitis vinifera* | VvMYB5b  VvMYBA1  VvMYBA2  VvMYBC2-L1 | AY899404  XM_003631456  AB097924  JX050227 |
| *Zea mays* | ZmP1  ZmC1  ZmMYB31  ZmMYB42 | L19495  AF320614  NM_001112479  NM_001112539 |
