# Supplementary material for: Transcriptome and Biochemical Analysis of a Flower Color Polymorphism in Silene littorea (Caryophyllaceae)
Source: Front Plant Sci. 2016 Feb 29;7:204. doi: 10.3389/fpls.2016.00204 (PMC4770042; doi:10.3389/fpls.2016.00204)
Supplement: Supplementary file 2 [file Table2.DOCX]

**Table S2. Flavonoid standards for HPLC analysis.** Standards used for identifying and quantifying flavonoids in the HPLC multiple reaction monitoring experiments, their mass transitions, and the references in which the compound derivatives were identified in other species of *Silene*.

| **Flavonoid Type** | **Standard** | **Mass transitions** | **References** |
| --- | --- | --- | --- |
| Anthocyanin | Peonidin | 301.1/301.1 | 1 (P) |
| Anthocyanin | Pelargonidin | 271.1/271.1 | 2 (P) |
| Anthocyanin | Cyanidin^a^ | 287.1/287.1 | 1, 2 (P) |
| Flavanone | Naringenin | 271.0/119.0 | - |
| Flavone | Apigenin^a^ | 269.0/117.0 | 3, 4, 5, 6, 7 (P, L) |
| Flavone | Apigenin 7-*O*-glucoside^a^ | 431.0/268.0 | 3, 4, 5, 6, 7 (P, L) |
| Flavone | Luteolin^a^ | 285.0/133.0 | 3, 5, 7 (P, L) |
| Flavonol | Kaempferol | 285.0/117.0 | 3, 4 (L) |
| Flavonol | Quercetin^a^ | 301.0/151.0 | 3, 4 (L) |
| Flavonol | Quercetin 3-*O*-rutinoside^a,c^ | 609.0/300.0 | - |
| Flavanonols | Dihydroquercetin^b,c^ | 303.0/303.0 | - |
| Isoflavone | Genisteine | 269.0/133.0 | 4 (L) |

^a^ compound derivative was confirmed in *S. littorea*

^b^ compound was only observed as trace in *S. littorea*

^c^ compound first time confirmed in species of *Silene*

**Table References**

1. Kuwayama S, Mori S, Nakata M, Godo T, Nakano M: **Analyses of anthocyanidins and anthocyanins in flower petals of *Lychnis senno* and its related species (Caryophyllaceae)**. *Bull Facul Agric Niigata Univ* 2005, **58**:35–38.

2. Kamsteeg J, van Brederode J, van Nigtevecht G: **Identification, properties, and genetic control of UDP-glucose: cyanidin-3-rhamnosyl-(1→ 6)-glucoside-5-O-glucosyltransferase isolated from petals of the red campion (*Silene dioica*).** *Biochem Genet* 1978, **16**:1059–1071.

3. Richardson M: **Flavonols and C-Glycosylflavonoids of the Caryophyllales**. *Biochem Syst Ecol* 1978, **6**:283–286.

4. Van de Staaij JWM, Ernst WHO, Hakvoort HWJ, Rozema J: **Ultraviolet-B (280–320 nm) absorbing pigments in the leaves of *Silene vulgaris*: their role in UV-B tolerance.** *J Plant Physiol* 1995, **147**:75–80.

5. Darmograi VN: **Flavonoids of plants of the genera *Silene* and *Otites adans*, family Caryophyllaceae**. *Chem Nat Comp* 1977, **13**:102–103.

6. Van Brederode J, Van Genderen HH, Berendsen W: **Morphological effects of the flavone isovitexin in a non-glycosylating genotype of *Silene pratensis* (Caryophyllaceae).** *Experientia* 1982, **38**:929–931.

7. Mastenbroek O, Rederode JV: **The possible evolution of *Silene pratensis* as deduced from present day variation patterns**. *Biochem Syst Ecol* 1986, **14**:165–181.
