# Supplementary material for: Transcriptome and Biochemical Analysis of a Flower Color Polymorphism in Silene littorea (Caryophyllaceae)
Source: Front Plant Sci. 2016 Feb 29;7:204. doi: 10.3389/fpls.2016.00204 (PMC4770042; doi:10.3389/fpls.2016.00204)
Supplement: Supplementary file 3 [file Table3.DOCX]

**Table S3. *Ans* SNP characterization.** Characteristics of the 32 SNPs found in the 5’UTR, coding sequence (CDS) and 3’ UTR of *Ans*. SNPs with color-differentiating allele frequencies are indicated in bold. W = white , L = light pink, D = dark pink.

| **Gene region** | **Alignment position** | **Reference allele** | **Alternate allele** | **Synonimous (S) or Non-synonimous (NS)** | **Alternate allele frequency in W** | **Alternate allele frequency in L** | **Alternate allele frequency in D** |
| --- | --- | --- | --- | --- | --- | --- | --- |
| 5' UTR | 86 | G | A |  | 0.000 | 0.500 | 0.333 |
| 5' UTR | 98 | A | C |  | 0.000 | 0.500 | 0.333 |
| CDS | 140 | G | A,C | NS | 0.167 | 0.833 | 0.167 |
| CDS | 179 | C | G | NS | 0.167 | 0.500 | 0.333 |
| CDS | 218 | C | T | S | 0.167 | 0.500 | 0.333 |
| CDS | 293 | G | C | NS | 0.333 | 0.667 | 0.333 |
| CDS | 305 | A | G | NS | 0.167 | 0.333 | 0.333 |
| CDS | 346 | C | G | NS | 0.167 | 0.667 | 0.333 |
| CDS | 403 | G | C | NS | 0.167 | 0.333 | 0.333 |
| CDS | 643 | T | C | S | 0.000 | 0.167 | 0.000 |
| **CDS** | **697** | **A** | **C** | **S** | **0.000** | **0.667** | **0.833** |
| CDS | 709 | A | G | S | 0.000 | 0.333 | 0.000 |
| **CDS** | **746** | **C** | **T** | **S** | **0.000** | **0.667** | **0.833** |
| **CDS** | **763** | **C** | **A** | **S** | **0.000** | **0.333** | **0.833** |
| **CDS** | **799** | **G** | **A** | **S** | **0.000** | **0.333** | **0.833** |
| CDS | 845 | C | T | S | 0.167 | 0.333 | 0.000 |
| CDS | 853 | T | C | S | 0.167 | 0.167 | 0.000 |
| **CDS** | **898** | **C** | **G** | **S** | **0.000** | **0.500** | **0.833** |
| **CDS** | **913** | **A** | **G** | **S** | **0.167** | **0.500** | **0.833** |
| CDS | 919 | C | T | S | 0.167 | 0.000 | 0.000 |
| **CDS** | **937** | **C** | **A** | **S** | **0.000** | **0.500** | **0.833** |
| CDS | 970 | T | C | S | 0.167 | 0.000 | 0.000 |
| CDS | 982 | C | T | S | 0.000 | 0.333 | 0.000 |
| **CDS** | **994** | **G** | **T** | **S** | **0.167** | **0.833** | **0.833** |
| **CDS** | **1099** | **A** | **G** | **S** | **0.167** | **0.667** | **0.667** |
| CDS | 1187 | C | T | NS | 0.167 | 0.167 | 0.000 |
| 3' UTR | 1301 | T | C |  | 0.167 | 0.167 | 0.000 |
| 3' UTR | 1311 | C | T |  | 0.167 | 0.167 | 0.000 |
| 3' UTR | 1343 | C | G |  | 0.000 | 0.333 | 0.500 |
| 3' UTR | 1352 | T | C |  | 0.167 | 0.167 | 0.000 |
| 3' UTR | 1360 | A | T |  | 0.167 | 0.167 | 0.000 |
| 3' UTR | 1428 | T | A |  | 0.167 | 0.000 | 0.500 |
