# Supplementary material for: Transcriptome and Biochemical Analysis of a Flower Color Polymorphism in Silene littorea (Caryophyllaceae)
Source: Front Plant Sci. 2016 Feb 29;7:204. doi: 10.3389/fpls.2016.00204 (PMC4770042; doi:10.3389/fpls.2016.00204)
Supplement: Supplementary file 4 [file Table4.DOC]

**Table S4. Expression differences between color morphs across developmental stages**. Expression corrected values and pairwise fold-change comparisons among color morphs and developmental stages for the 29 ABP-related loci. In bold are highlighted those pairwise comparisons that are significant (p<0.05) from the negative binomial tests.

| **BUD EXPRESSION** | | | | | | |
| --- | --- | --- | --- | --- | --- | --- |
|  | **Corrected expression values** | | | **Fold change** | | |
| **Loci** | **Dark** | **Light** | **White** | **D/W** | **L/W** | **D/L** |
| *Pal1* | 11227.9 | 11376.8 | 7880.8 | 1.4 | 1.4 | 1.0 |
| *Pal2* | 494875.6 | 354991.8 | 104448.7 | 4.7 | 3.4 | 1.4 |
| *Pal3* | Filtered | Filtered | Filtered | - | - | - |
| *C4h1* | 37635.7 | 28535.9 | 16466.5 | 2.3 | 1.7 | 1.3 |
| *C4h2* | 52874.9 | 13603.8 | 1459.8 | **36.2** | 9.3 | 3.9 |
| *4Cl1* | Filtered | Filtered | Filtered | - | - | - |
| *4Cl2* | Filtered | Filtered | Filtered | - | - | - |
| *Chs1* | 6094.3 | 21439.8 | 25948.1 | 0.2 | 0.8 | 0.3 |
| *Chs2* | 2087.1 | 4420.8 | 8713.9 | 0.2 | 0.5 | 0.5 |
| *Chi* | 10885.0 | 4319.7 | 13372.6 | 0.8 | 0.3 | 2.5 |
| *F3h1* | 73300.0 | 63067.7 | 1495.7 | **49.0** | **42.2** | 1.2 |
| *F3h2* | Filtered | Filtered | Filtered | - | - | - |
| *F3h3* | Filtered | Filtered | Filtered | - | - | - |
| *Dfr* | 8568.6 | 12976.5 | 8607.5 | 1.0 | 1.5 | 0.7 |
| *Ans* | 13372.4 | 14939.3 | 11138.7 | 1.2 | 1.3 | 0.9 |
| *Uf3gt* | 7360.6 | 11363.1 | 27497.6 | 0.3 | 0.4 | 0.6 |
| *At* | 8529.3 | 6460.1 | 7227.2 | 1.2 | 0.9 | 1.3 |
| *F3'h* | 5613.5 | 8889.9 | 1969.9 | 2.8 | **4.5** | 0.6 |
| *Fls* | Filtered | Filtered | Filtered | - | - | - |
| *Myb1a* | 21442.0 | 5085.6 | 4171.7 | **5.1** | 1.2 | **4.2** |
| *Myb1b* | 4394.4 | 5782.2 | 8496.0 | 0.5 | 0.7 | 0.8 |
| *Myb2* | 5423.4 | 7756.6 | 6723.5 | 0.8 | 1.2 | 0.7 |
| *Myb3* | 3152.2 | 10583.5 | 5140.7 | 0.6 | 2.1 | **0.3** |
| *Myb4* | Filtered | Filtered | Filtered | - | - | - |
| *Myb5* | 16931.9 | 12480.6 | 15756.5 | 1.1 | 0.8 | 1.4 |
| *Myb6* | 4175.2 | 7195.7 | 1530.3 | 2.7 | 4.7 | 0.6 |
| *Wd401* | Filtered | Filtered | Filtered | - | - | - |
| *Wd402* | Filtered | Filtered | Filtered | - | - | - |
| *Bhlh* | Filtered | Filtered | Filtered | - | - | - |
| **OPENING EXPRESSION** | | | | | | |
|  | **Corrected expression values** | | | **Fold change** | | |
| **Loci** | **Dark** | **Light** | **White** | **D/W** | **L/W** | **L/D** |
| *Pal1* | 13899.1 | 17384.5 | 29031.4 | 0.5 | 0.6 | 0.8 |
| *Pal2* | 181519.5 | 70311.2 | 219069.5 | 0.8 | 0.3 | 2.6 |
| *Pal3* | 2781.2 | 574.1 | 5732.4 | 0.5 | 0.1 | 4.8 |
| *C4h1* | 54156.6 | 129645.7 | 131416.0 | 0.4 | 1.0 | 0.4 |
| *C4h2* | 11208.5 | 16123.7 | 5256.7 | 2.1 | 3.1 | 0.7 |
| *4Cl1* | 2048.3 | 13119.7 | 5439.1 | 0.4 | 2.4 | 0.2 |
| *4Cl2* | Filtered | Filtered | Filtered | - | - | - |
| *Chs1* | 64507.1 | 17417.9 | 95468.0 | 0.7 | 0.2 | 3.7 |
| *Chs2* | 6067.0 | 5249.6 | 2730.8 | 2.2 | 1.9 | 1.2 |
| *Chi* | 18093.4 | 7847.8 | 14507.5 | 1.2 | 0.5 | 2.3 |
| *F3h1* | 55358.2 | 108048.4 | 34653.6 | 1.6 | 3.1 | 0.5 |
| *F3h2* | Filtered | Filtered | Filtered | - | - | - |
| *F3h3* | Filtered | Filtered | Filtered | - | - | - |
| *Dfr* | 6176.6 | 7508.8 | 271.6 | **22.7** | **27.6** | 0.8 |
| *Ans* | 10488.3 | 4911.9 | 2507.3 | 4.2 | 2.0 | 2.1 |
| *Uf3gt* | 5816.1 | 2765.2 | 887.0 | 6.6 | 3.1 | 2.1 |
| *At* | 26617.0 | 12866.1 | 15538.9 | 1.7 | 0.8 | 2.1 |
| *F3'h* | 8397.1 | 8274.7 | 10390.8 | 0.8 | 0.8 | 1.0 |
| *Fls* | Filtered | Filtered | Filtered | - | - | - |
| *Myb1a* | 4084.0 | 10942.2 | 12233.0 | 0.3 | 0.9 | 0.4 |
| *Myb1b* | Filtered | Filtered | Filtered | - | - | - |
| *Myb2* | 5157.0 | 7605.3 | 3473.4 | 1.5 | 2.2 | 0.7 |
| *Myb3* | Filtered | Filtered | Filtered | - | - | - |
| *Myb4* | Filtered | Filtered | Filtered | - | - | - |
| *Myb5* | 9424.4 | 15265.1 | 14868.4 | 0.6 | 1.0 | 0.6 |
| *Myb6* | 6432.4 | 29972.2 | 19830.2 | 0.3 | 1.5 | **0.2** |
| *Wd401* | Filtered | Filtered | Filtered | - | - | - |
| *Wd402* | Filtered | Filtered | Filtered | - | - | - |
| *Bhlh* | Filtered | Filtered | Filtered | - | - | - |
| **ANTHESIS EXPRESSION** | | | | | | |
|  | **Corrected expression values** | | | **Fold change** | | |
| **Loci** | **Dark** | **Light** | **White** | **D/W** | **L/W** | **D/L** |
| *Pal1* | 19718.7 | 26183.0 | 30892.6 | 0.6 | 0.8 | 0.8 |
| *Pal2* | 244895.1 | 246964.6 | 159794.4 | 1.5 | 1.5 | 1.0 |
| *Pal3* | 3893.3 | 9419.8 | 2831.1 | 1.4 | 3.3 | 0.4 |
| *C4h1* | 124206.3 | 131175.3 | 129845.9 | 1.0 | 1.0 | 0.9 |
| *C4h2* | 14706.6 | 9413.2 | 8742.8 | 1.7 | 1.1 | 1.6 |
| *4Cl1* | 7593.1 | 3005.9 | 2701.1 | 2.8 | 1.1 | 2.5 |
| *4Cl2* | Filtered | Filtered | Filtered | - | - | - |
| *Chs1* | 49619.5 | 106225.9 | 16991.9 | 2.9 | **6.3** | 0.5 |
| *Chs2* | 4507.4 | 17097.7 | 1066.3 | 4.2 | 16.0 | 0.3 |
| *Chi* | 19249.6 | 22681.9 | 13365.0 | 1.4 | 1.7 | 0.8 |
| *F3h1* | 82269.0 | 34089.9 | 142819.6 | 0.6 | 0.2 | 2.4 |
| *F3h2* | Filtered | Filtered | Filtered | - | - | - |
| *F3h3* | Filtered | Filtered | Filtered | - | - | - |
| *Dfr* | 5457.7 | 5294.4 | 3419.4 | 1.6 | 1.5 | 1.0 |
| *Ans* | 13391.8 | 8980.8 | 9763.1 | 1.4 | 0.9 | 1.5 |
| *Uf3gt* | 6259.6 | 6572.6 | 2782.5 | 2.2 | 2.4 | 1.0 |
| *At* | 4257.1 | 2000.5 | 13966.4 | 0.3 | 0.1 | 2.1 |
| *F3'h* | 11068.4 | 17520.6 | 14428.6 | 0.8 | 1.2 | 0.6 |
| *Fls* | Filtered | Filtered | Filtered | - | - | - |
| *Myb1a* | 7899.3 | 8972.2 | 16273.6 | 0.5 | 0.6 | 0.9 |
| *Myb1b* | Filtered | Filtered | Filtered | - | - | - |
| *Myb2* | 2233.4 | 2706.6 | 6476.3 | 0.3 | 0.4 | 0.8 |
| *Myb3* | Filtered | Filtered | Filtered | - | - | - |
| *Myb4* | Filtered | Filtered | Filtered | - | - | - |
| *Myb5* | 12226.0 | 16661.5 | 10346.1 | 1.2 | 1.6 | 0.7 |
| *Myb6* | 4877.6 | 4534.1 | 25960.5 | 0.2 | **0.2** | 1.1 |
| *Wd401* | Filtered | Filtered | Filtered | - | - | - |
| *Wd402* | Filtered | Filtered | Filtered | - | - | - |
| *Bhlh* | Filtered | Filtered | Filtered | - | - | - |
