# Supplementary material for: Transcriptome and Biochemical Analysis of a Flower Color Polymorphism in Silene littorea (Caryophyllaceae)
Source: Front Plant Sci. 2016 Feb 29;7:204. doi: 10.3389/fpls.2016.00204 (PMC4770042; doi:10.3389/fpls.2016.00204)
Supplement: Supplementary file 5 [file Table5.DOCX]

**Table S5.** **Flavonoid identification**. Results from the HPLC–ESI-MS/MS biochemical analysis of petals with flavonoid identifications.

| **Flavonoid type** | **Flavonoid identification^b^** | ***t_R_* (min)** | **Parent ions (*m/z*)** | **MS/MS (*m/z*)^a^** |
| --- | --- | --- | --- | --- |
| Anthocycanin | Cyanidin acetylrutinoside-glucoside | 4.00 | 800 | 637/449/287 |
| Anthocycanin | Cyanidin 3-*O*-rutinoside-5-*O*-glucoside | 4.57 | 757 | 595/449/287 |
| Anthocycanin | Cyanidin acetylrutinoside-glucoside | 5.70 | 800 | 637/449/287 |
| Dihydroflavonol | Dihydroquercetin^c^ | 10.80 | 303 | 303 |
| Flavone | Isoorientin | 6.00 | 447 | 285/133 |
| Flavone | Isovitexin | 9.97 | 431 | 240 |
| Flavone | Luteolin | 10.84 | 285 | 133 |
| Flavone | Apigenin | 11.30 | 269 | 117 |
| Flavonol | Rutin | 9.48 | 609 | 300 |
| Flavonol | Quercetin | 10.94 | 301 | 151 |

^a^ MS analysis was acquired in positive mode for anthocyanins and in negative mode for non-anthocyanin flavonoids; ^b^ identification was based on retention time and comparisons of MS data with standards and values of previously reported flavonoids for *Silene* species (see Table S2 and the database http://metabolomics.jp); ^c^ this compound was observed as trace.
