# Supplementary material for: Transcriptome and Biochemical Analysis of a Flower Color Polymorphism in Silene littorea (Caryophyllaceae)
Source: Front Plant Sci. 2016 Feb 29;7:204. doi: 10.3389/fpls.2016.00204 (PMC4770042; doi:10.3389/fpls.2016.00204)
Supplement: Supplementary file 6 [file Image1.PDF]

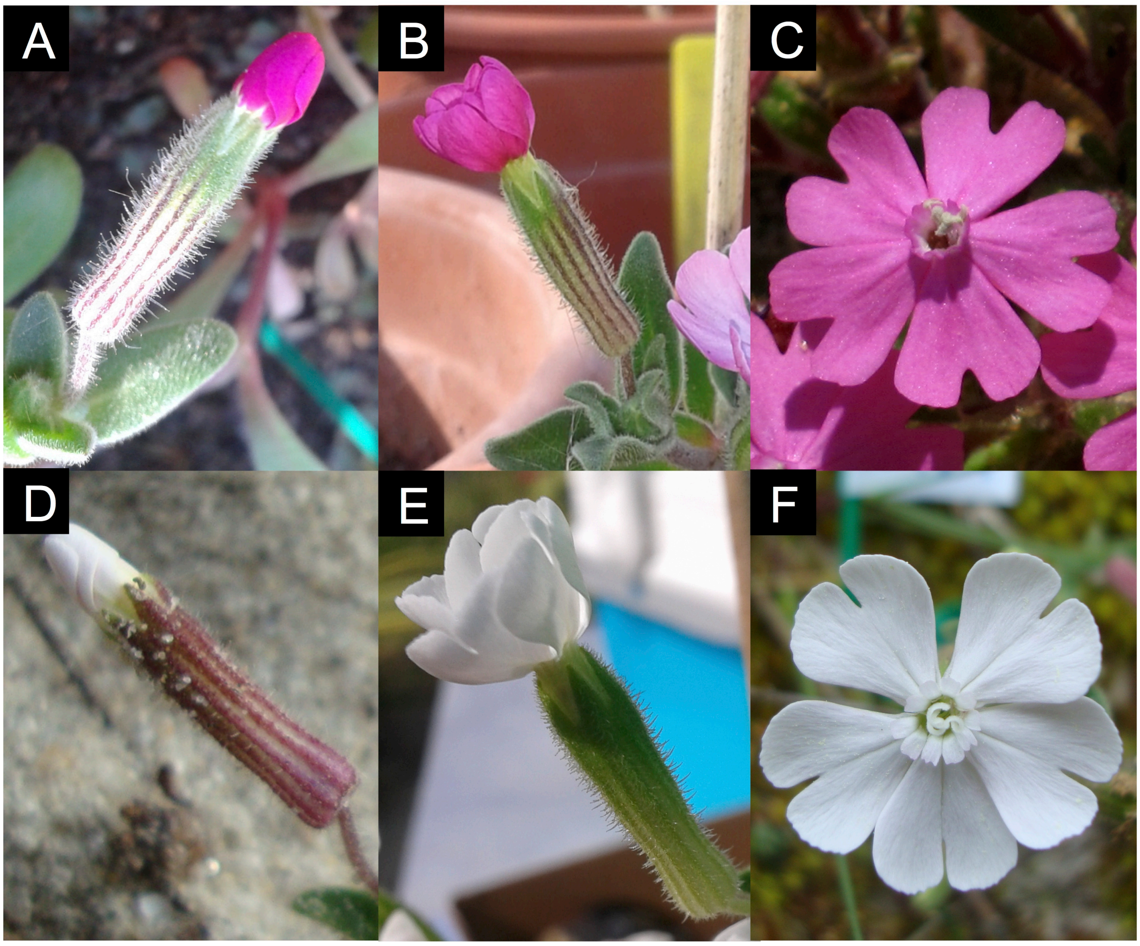

Figure S1. Developmental differences in dark pink and white petals.
Images of the three developmental stages sampled for dark pink and white flower color morphs of *Silene littorea*: bud (A, D), opening (B, E), and anthesis (C, F).
