# Supplementary material for: Transcriptome and Biochemical Analysis of a Flower Color Polymorphism in Silene littorea (Caryophyllaceae)
Source: Front Plant Sci. 2016 Feb 29;7:204. doi: 10.3389/fpls.2016.00204 (PMC4770042; doi:10.3389/fpls.2016.00204)
Supplement: Supplementary file 7 [file Image2.PDF]

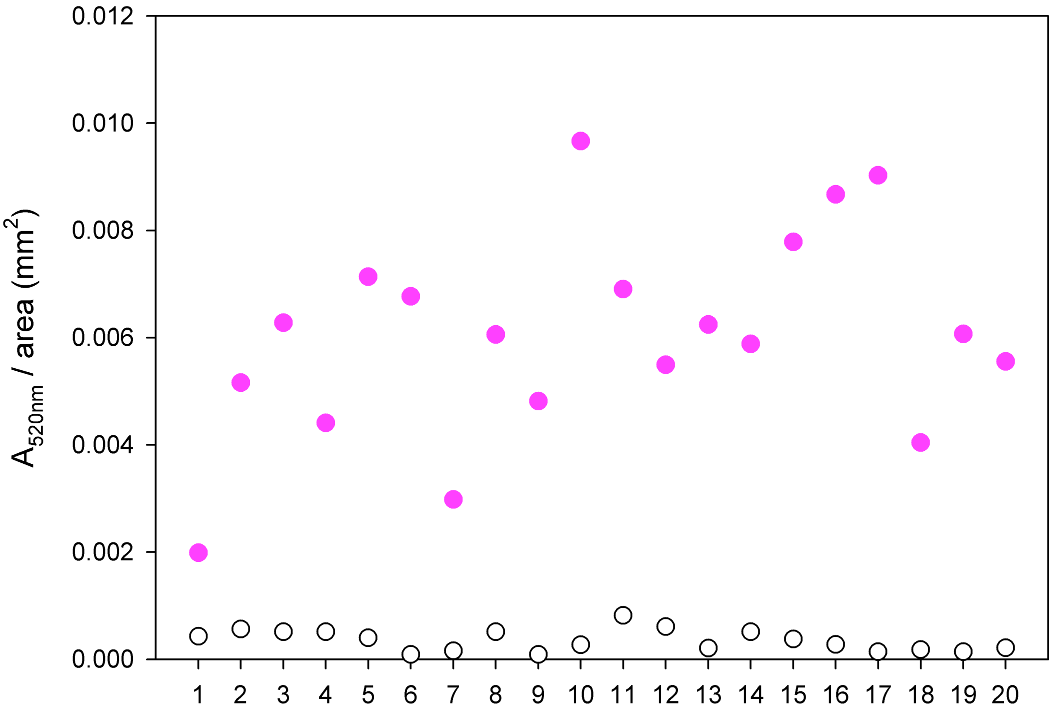

Figure S2. Absorbance values of pink (pink circles) and white samples (white circles) used for ANS Sanger sequencing. Absorbance values are corrected by area (mm^2).
