# Supplementary material for: Transcriptome and Biochemical Analysis of a Flower Color Polymorphism in Silene littorea (Caryophyllaceae)
Source: Front Plant Sci. 2016 Feb 29;7:204. doi: 10.3389/fpls.2016.00204 (PMC4770042; doi:10.3389/fpls.2016.00204)
Supplement: Supplementary file 8 [file Image3.PDF]

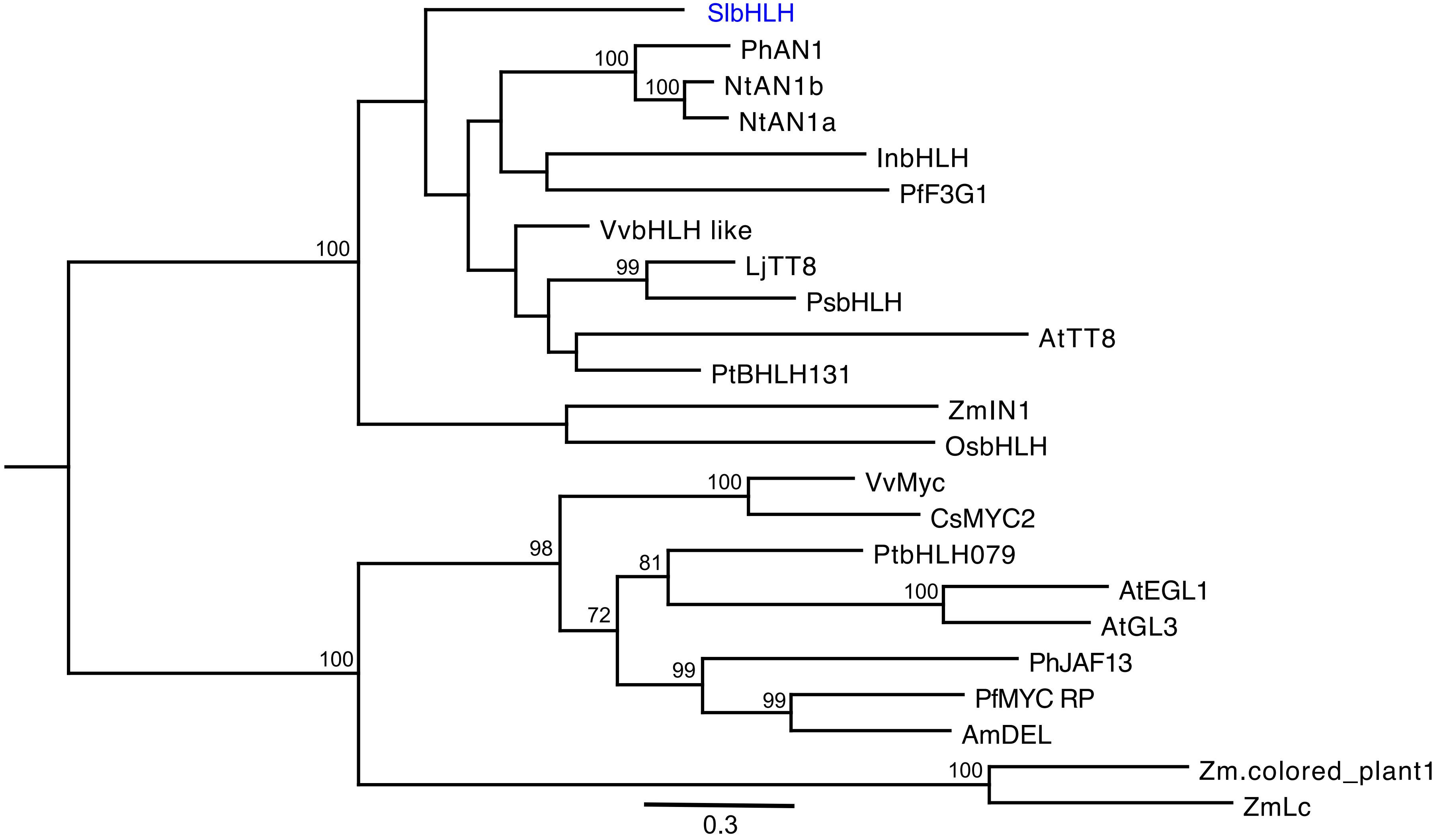

Figure S3. Phylogenetic analysis of bHLH aminoacid sequences for *S. littorea* and other model species. We conducted a maximum likelihood (RAxML; Stamatakis, 2014) phylogenetic analysis of the aligned aminoacids using plug-ins within Geneious v.8.1.6. We fit a GAMMA BLOSUM62 model followed by 1,000 bootstrap replications. Accession numbers and abbreviations: AmDEL (*Antirrhinum majus*, AAA32663.1), AtGL3 (*Arabidopsis thaliana*, NP_680372.1), AtEGL1 (*A. thaliana*, NP_176552.1), AtTT8 (*Arabidopsis thaliana*, CAC14865.1), CsMYC2 (*Citrus sinensis*, ABR68793.1), InbHLH (*Ipomoea nil*, BAE94394.1), LjTT8 (*Lotus japonicus*, BAH28881.1), NtAN1a (*Nicotiana tabacum*, AEE99257.1), NtAN1b (*N. tabacum*, AEE99258.1), OsbHLH (*Oryza sativa Indica Group*, BAF42668.1), PfMYC-RP (*Perilla frutescens*, BAA75513.1), PfF3G1 (*P. frutescens*, BAC56998.1), PhAN1(*Petunia x hybrida*, AF260918_1), PhJAF13 (*Petunia x hybrida*, AAC39455.1), PsbHLH (*Pisum sativum*, ADO13282.1), PtbHLH079 (*Populus trichocarpa*, XP_002299530.1), PtbHLH131 (*P. trichocarpa*, Potri.005G208600.1), SlbHLH (*Silene littorea*, VvMyc (*Vitis vinifera*, ABM92332.3), VvbHLH-like (*V. vinifera*, ACC68685.1), ZmIN1 (*Zea mays*, AAB03841.1), ZmLc (*Z. mays*, NP_001105339.1), Zm.colored plant1 (*Zea mays*, NP_001105706.1)
