# Supplementary material for: Transcriptome and Biochemical Analysis of a Flower Color Polymorphism in Silene littorea (Caryophyllaceae)
Source: Front Plant Sci. 2016 Feb 29;7:204. doi: 10.3389/fpls.2016.00204 (PMC4770042; doi:10.3389/fpls.2016.00204)
Supplement: Supplementary file 9 [file Image4.PDF]

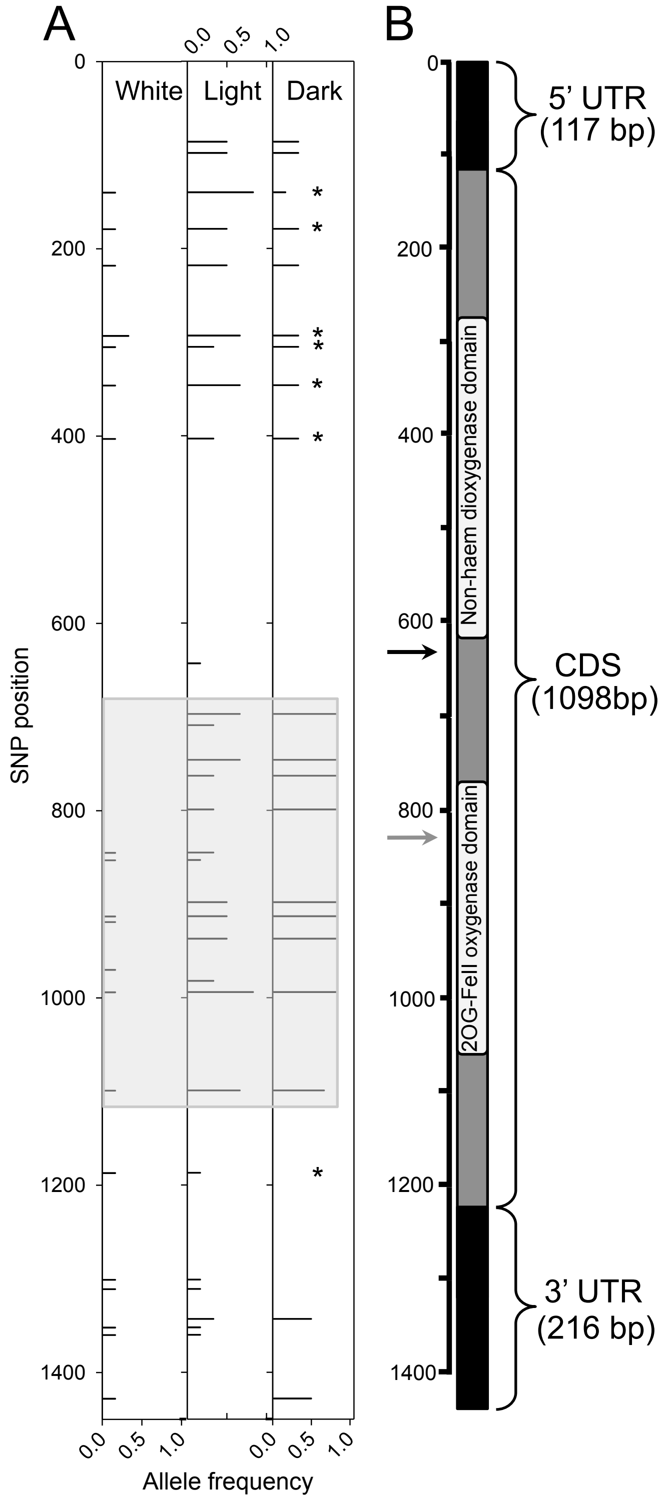

Figure S4. Allele frequencies of the SNPs found in the *Ans* gene and characterization of the gene regions. Allele frequencies of the SNPs in *Ans* (A) relative to the main functional domains of the gene (B). In (A) the grey box highlights the region of nine synonymous SNPs that correlate with flower color (between bp 697 and 1100). Asterisks indicate non-synonymous SNPs. In (B), the gene is composed of a 5' UTR, 3' UTR and CDS containing two functional domains. The location of the intron is indicated with a black arrow in (B). The grey arrow indicates the inferred position of the iron binding domain.
