# Supplementary material for: Transcriptome and Biochemical Analysis of a Flower Color Polymorphism in Silene littorea (Caryophyllaceae)
Source: Front Plant Sci. 2016 Feb 29;7:204. doi: 10.3389/fpls.2016.00204 (PMC4770042; doi:10.3389/fpls.2016.00204)
Supplement: Supplementary file 10 [file Image5.PDF]

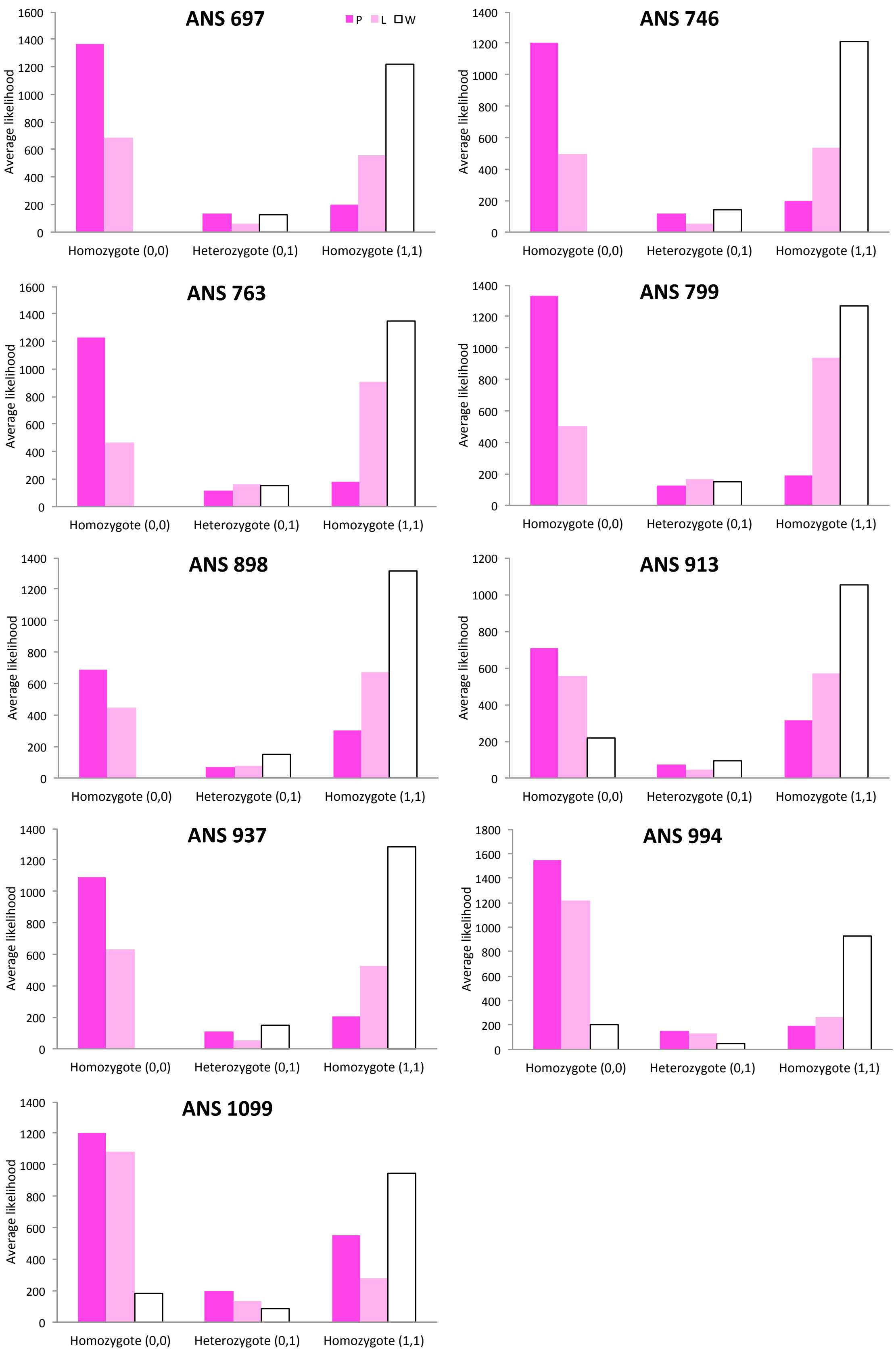

Figure S5. Genotype likelihoods for the nine synonymous SNPs in Ans that correlate with flower color. The likelihood of genotype assignments for homozygotes for the reference allele (0,0), homozygotes for the alternate allele (1,1) and heterozygotes (0,1) for dark pink (D), light pink (L) and white (W) samples from GATK. Alignment position is indicated above each graph.
